# Long‐lasting housing environment manipulation and acute loss of environmental enrichment impact BALB/c mice behaviour in multiple functional domains

**DOI:** 10.1111/ejn.15602

**Published:** 2022-02-23

**Authors:** Momoe Sukegawa, Toru Yoshihara, Shengqun Hou, Masahide Asano, Anthony J. Hannan, Dan Ohtan Wang

**Affiliations:** ^1^ Center for Biosystems Dynamics Research (BDR), RIKEN Kobe Japan; ^2^ Graduate School of Biostudies Kyoto University Kyoto Japan; ^3^ Institute for Integrated Cell‐Material Sciences (iCeMS) Kyoto University Kyoto Japan; ^4^ Institute of Laboratory Animals, Graduate School of Medicine Kyoto University Kyoto Japan; ^5^ Department of Anatomy and Neuroscience University of Melbourne Parkville Victoria Australia; ^6^ Melbourne Brain Centre Parkville Victoria Australia

**Keywords:** BALB/c, behavioural test battery, enriched environment, enrichment removal, social isolation

## Abstract

Understanding environmental influences on individuals' behaviour is challenging. Here we have investigated the housing impact of 9 weeks of enriched environment (EE) and social isolation (SI) and the impact of abrupt deprivation of EE (enrichment removal: ER) on BALB/c mice. Compared with the widely used C57BL/6 strain in research, BALB/c synthesises serotonin less efficiently due to a genetic variation and thus may potentially represent human populations at higher risk of stress‐related disorders. We assessed the effects of EE and SI by conducting a behavioural test battery and the effects of acute ER by monitoring homecage activities and social behaviour. We found that EE and SI impact BALB/c's physiological states and behavioural performances from lower to higher cognitive processes: increased body weight, increased rectal temperature, altered performance in motor and sensory tasks, the activity level in a novel environment and altered performance in tests of anxiety‐like behaviour, stress‐coping strategies and learning and memory. Furthermore, acute ER triggered stress/frustration‐like behaviour in BALB/c, with increased aggression, increased social distancing and disrupted daily/nightly activities. Our results demonstrate that long‐lasting housing manipulation such as EE and SI, impact behaviour via multilayered processes over a wide range of functional domains, and unforeseen change to a negative environment, ER, is a major stressor that causes behavioural and psychological consequences through environment–gene interactions, a model of direct relevance to human health.

## Graphical abstract

- Long‐lasting housing environment manipulation impact physiological states and behaviour of BALB/c mice in multiple functional domains.
- Acute enrichment removal triggers aggression, social distancing, and daily/nightly activity disruption.
- Behaviour elicited by acute enrichment removal is reminiscent of major stressor imposition.

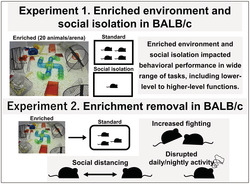

**Abbreviations**
EE: enriched environment
ER: enrichment removal
SI: social isolation
ST: standard housing

## 1 INTRODUCTION

Environmental factors critically contribute to shaping our cognitive and emotional functions because our connections to the external world help build, maintain and modify our biological/psychological systems (Briley & Tucker‐Drob, 2014; Dick, 2011). Elucidating the process of how environmental factors influence our behaviour will help us better understand the processes of personality development and predisposition to brain disorders, thus revealing opportunities for new educational and therapeutic approaches. However, delineating the biological and psychological links between specific environmental influences and an individual's behaviour remains extremely challenging (Baumert et al., 2017; Lambert et al., 2019; Yap & Greenberg, 2018).

Rodents allow researchers to use carefully controlled environmental and invasive approaches and thus serve as valuable animal models for studying human behaviour and disorders (Richter‐Levin et al., 2019). In laboratory standard housing (ST) conditions, rodents are usually housed in ‘shoebox’ cages with a few cage mates. This housing environment can be enriched or deprived of sensory and/or social stimuli. Enriched environment (EE) housing for laboratory rodents often consists of a variety of toys, running wheels and a larger number of cage mates in larger‐sized cages, although EE protocols vary widely. EE provides animals with opportunities to experience quantitatively more abundant and qualitatively more complex stimuli over multiple modalities. EE has been shown to enhance learning and memory, decrease anxiety‐like behaviour, facilitate motor function and alter communication patterns in animals, for example, increased fighting behaviour (Gubert & Hannan, 2019; Kempermann, 2019; McQuaid et al., 2012; Nithianantharajah & Hannan, 2006). The therapeutic potential of EE in neurodegenerative and other brain diseases has been investigated; one of the underlying mechanisms is enhanced adult neurogenesis (Martínez‐Cué et al., 2002; Nithianantharajah & Hannan, 2006; Restivo et al., 2005; van Dellen et al., 2000).

In contrast to the generally positive effects of EE, social isolation (SI) (lacking all or partial social relationships) has been shown to have profoundly negative effects on social animals, including humans. In humans, perceived SI is a risk factor for poorer cognitive performance, executive function, negativity, depressive cognition, increased social threats and self‐protective biases (Cacioppo & Hawkley, 2009). In laboratory SI housing conditions, animals are housed in standard cages singly for continuous periods of time with no opportunities for social contact. Social component of the housing environment strongly impacts mice (Gómez et al., 2017), and SI can result in hyperactivity and increased anxiety‐like behaviour in animals with the disrupted function of monoamine systems (Walker et al., 2019).

Sudden negative environmental changes represent another stressful situation. Life events related to ‘loss’ are one of the most serious stressors in human society (Holmes & Rahe, 1967). Recently, Morano et al. (2019) and Smith et al. (2017) exposed rats to EE and subsequently transferred them to ST, thus enrichment removal (ER). These animals showed increased immobility in the Porsolt swim test interpreted as a depressive‐like behaviour. Thus, the ER paradigm can be potentially used for studying the psychological effect of negative environmental changes. However, the behavioural effect of ER is underexplored, especially from the aspects of social domains despite their scientific and societal importance.

Here we have focused on the effects of EE, SI and ER on the behaviour of BALB/c mice. Previous studies of housing manipulation have paid attention to emotion, learning and memory. In the present study, in addition to these domains, we have also included sensory function, motor function and activity levels in behavioural assessment. This approach renders it possible to consider the involved multiple domains in producing the behaviour. Furthermore, by conducting a 2‐week ER and video tracking the interactions between individuals in home‐cages, we assessed the effects of ER on sociality. BALB/c mice are known as a behaviourally sensitive strain to the surrounding environment (Francis et al., 2003) and thus could serve as a model system for sensitive human populations. However, only handful studies have been published regarding the impact of housing manipulation on the behaviour of BALB/c mice. Chapillon et al. (1999) and Roy et al. (2001) reported decreased anxiety‐like behaviour and activity level in a novel environment provided by EE in BALB/c mice. Du Preez et al. (2020) and An et al. (2017) reported increased anxiety‐like behaviour, depressive‐like behaviour, cognitive deficits and aggressive behaviour induced by SI in BALB/c mice. The characteristic sensitivity of BALB/c mice to environments is postulated to be partially related to single‐nucleotide polymorphism (SNP) C1473G in tryptophan hydroxylase 2 (Tph2) gene. TPH2 catalyses the first step of brain serotonin synthesis (Zhang et al., 2004). Homozygous 1473G alleles in BALB/c result in lower TPH2 activity than in strains expressing the 1473C alleles (Osipova et al., 2009). In the current study, we have investigated the behavioural effect of long‐lasting EE and SI in BALB/c mice on a wide range of functional domains by conducting a behavioural test battery including tests of general health state, sensory function, motor function, activity level, anxiety‐like behaviour, sociality, stress‐coping strategy and learning and memory. Moreover, we explored the social effect of acute ER by conducting a detailed video analysis of animals' behaviour and a behavioural test battery under ER.

## 2 MATERIALS AND METHODS

We have performed two experiments in the current study. In Experiment 1, we explored the effect of long‐lasting EE and SI in BALB/c mice. Animals were housed in ST, EE or SI from 3 weeks postnatal until the end of 11 weeks of age. Subsequently, the behavioural test battery was performed on these three groups. In Experiment 2, we explored the effect of acute ER on animals' social interaction patterns. Animals were housed in ST or EE from postnatal 3 weeks until the end of 11 weeks of age. Subsequently, both groups of animals were transferred to ST* (with similar numbers of cage mates and cage size to ST but equipped with video cameras) and kept in ST* for 14 days. Animals' activity level and social behaviour in ST*, especially aggressive behaviour, were measured. Subsequently, we conducted a behavioural test battery. Because of serious animal fighting, we limited the battery to open‐field, Crawley's social interaction and tail suspension tests from ethical perspectives.

### 2.1 Animals

Postnatal days 21–22 (P21–22) weaned male BALB/cCrSlc mice were purchased from Japan SLC (Shizuoka, Japan). Upon receival, animals were randomly assigned to each group for experiments. For stranger animals of the Crawley's social interaction test, postnatal 6‐ to 7‐week‐old male C57BL/6NCrSlc mice were purchased from Japan SLC (Shizuoka, Japan). The animal experiments were conducted in accordance with the Fundamental Guidelines for Proper Conduct of Animal Experiment and Related Activities in Academic Research Institutions under the jurisdiction of the Ministry of Education, Culture, Sports, Science and Technology of Japan approved by the Committee on Animal Experimentation of Kyoto University (#42‐5).

### 2.2 Housing

Animals were housed in specific‐pathogen‐free rooms with 12 h of light–dark cycle and fed ad libitum.

ST animals were reared in standard‐sized cages (W 234 mm, D 373 mm, H 140 mm, four mice/cage for Experiment 1, three mice/cage for Experiment 2, 220–290 cm^2^/mouse) from P21–22 until the end of 11 weeks of age. We reduced the number of animals to three per standard cage in Experiment 2 for better video tracking and behavioural monitoring. No environmental enrichment including hiding spaces was provided. EE animals were reared in open‐top arenas (see Figure S1, W 900 mm, D 1200 mm, H 450 mm, 20 mice/cage for Experiment 1, 23 mice/cage for Experiment 2, 470–540 cm^2^/mouse) from P21–22 until the end of 11 weeks of age. We followed Slater and Cao (2015) to set up objects in EE with additional wooden logs and metal mesh toys. In this protocol, EE has two cages inside to provide water and food to animals. Plastic toys (hollow balls, small arch shelters, small square shelters, big square shelters and two‐layered shelters) and saucer wheels were obtained from Bio‐Serv (New Jersey, USA). Plastic tubes (37–45 mm in diameter) and big metal running wheels were purchased from Sanko (Osaka, Japan). The objects in EE were rearranged and cleaned weekly. SI animals were reared in standard cages (W 234 mm, D 373 mm, H 140 mm, one mouse/cage, 870 cm^2^/mouse) from P21–22 until the end of 11 weeks of age. All groups of animals were housed with bedding materials made of white paper.

After the above housing manipulation, during the behavioural test battery in Experiment 1, ST animals and SI animals were housed in standard size cages (W 140 mm, D 265 mm, H 105 mm, four mice/cage for ST animals, one mouse/cage for SI animals). EE animals were housed in semi‐EE cages (W 310 mm, D 475 mm, H 295 mm, four mice/cage, 370 cm^2^/mouse) to proceed experimental procedure smoothly and avoid stressful situations from being chased by the experimenter in the wide EE arena during behavioural tests. Semi‐EE cages contained two saucer wheels, a feeding cage, one or two wooden logs and several plastic toys and tubes also used for EE (see Figure S1). The schedule to rearrange and clean objects in semi‐EE cages was the same to EE.

In Experiment 2, animals housed in ST and animals housed in EE were housed in ST* (different type standard cage; W 160 mm, D 265 mm, H 300 mm, three mice/cage, 140 cm^2^/mouse) with the different type bedding material (pellets of black recycled paper) and ceiling illumination for video recording (O'HARA, Tokyo, Japan). No environmental enrichment including hiding spaces was provided. Animals were housed in ST* during the behavioural test battery.

### 2.3 Procedure

**FIGURE 1:**
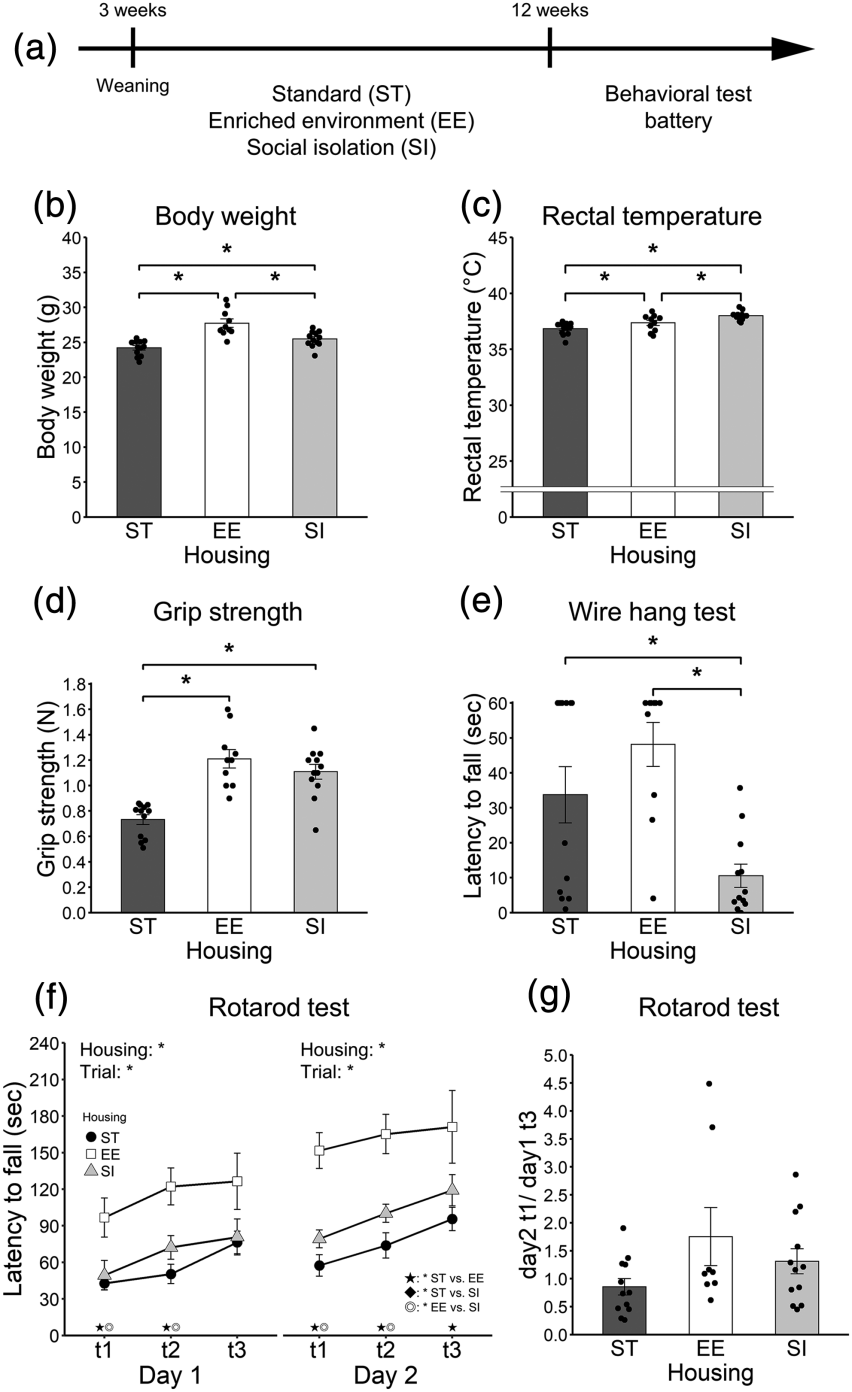
General health and motor function. (a) The schema of Experiment 1. (b) Body weight. (c) Rectal temperature. (d) Grip strength. (e) Latency to fall in the wire hang test. (f) Latency to fall in the rotarod test. (g) The ratio of performances of day 2 trial 1/day 1 trial 3 in the rotarod test. Error bars represent standard errors of the mean. Asterisks represent adjusted *p* < .05. ST: standard housing. EE: enriched environment. SI: social isolation

In Experiment 1, the behavioural test battery was conducted on ST, EE and SI animals (Figure 1a). This test battery included the open‐field, Y‐maze, light–dark box, elevated‐plus maze, rotarod, hot plate, Crawley's social interaction, Porsolt swim, prepulse inhibition, Barnes maze, fear‐conditioning and tail suspension tests (see Table S1). We performed one behavioural test or trial per day. General health and neurological examination were conducted at the beginning of the test battery, and all experiments were finished before the animals reached 26 weeks of age. The behavioural test battery was performed during the last 7 h of the light cycle. We excluded animals from tests when animals were ailing and not appropriate for conducting behavioural tests from ethical perspectives.

In Experiment 2, animals were transferred to ST* after 9 weeks of ST or EE housing and video recorded for cage activities in ST* 14 days, 24 h a day (Figure 8a). ST* cages were changed for cleaning in the light phase on day 6 and on day 13. At the beginning and end of ST*, individual animals were constrained in a transparent cylinder (25 mm in diameter) for a few seconds for their tails to be photographed on both dorsal and ventral sides. These photos were later analysed for counting wounds as physical evidence of animal fights. Subsequently, the select behavioural test battery was performed (the open‐field test, Crawley's social interaction test, and tail suspension test) after handling during the last 7 h of the light cycle (see Table S1). We performed one behavioural test or trial per day.

### 2.4 Behavioural experiments

#### 2.4.1 General health and neurological examination

General health and neurological examination were performed prior to the test battery. The first day test included rectal temperature, body weight, whisker state (trimmed or not?), coat state (injured, bald, or not?), righting reflex, whisker twitch, ear twitch, reaching and wire hang test. The second day tests included grip strength and epilepsy tests. See detailed methods in the supporting information.

#### 2.4.2 Open‐field test

The open‐field test was performed in an arena of W 407 mm, D 407 mm and H 305 mm illuminated at 100 lux (Accuscan Instruments, Ohio, USA), and animals could freely explore the arena for 30 min. Animals were positioned in the front left corner of the arena to start experiments. The total distance travelled, time spent in the centre area (inner 30% area) and the number of vertical activities were recorded. For analysis, we calculated the summation of locomotion activities in each 5‐min block.

#### 2.4.3 Y‐maze

The Y‐maze was performed in an apparatus (O'HARA, Tokyo, Japan) with three arms arranged at 120° intervals (length: 400 mm, height: 120 mm, lower bottom width: 30 mm, upper bottom width: 120 mm; illuminated at 100 lux) for 5 min. The total distance travelled and alternation rate (the number to enter all three arms within three entries/ [the total number of entries into arms] ‐2) were recorded.

#### 2.4.4 Light–dark box test

The light–dark box test was performed in an arena of W 405 mm, D 200 mm, H 249 mm (O'HARA, Tokyo, Japan), for 10 min. The arena was equally divided into two chambers separated by a wall: a light white‐coloured chamber with ceiling illumination (550 lux) and a dark black‐coloured chamber without illumination. The wall has a hole (W 50 mm, D 30 mm) that allowed animals to move freely between two chambers. Animals were positioned in the dark chamber when the experiment was started and allowed to move freely during the test. The total distance travelled, time spent in each chamber and the latency to enter the light chamber were recorded.

#### 2.4.5 Elevated‐plus maze

The elevated‐plus maze was performed in an arena (O'HARA, Tokyo, Japan) which has four arms (W 50 mm, D 250 mm, 550 mm above the floor; two with 2‐mm ledges and the other two with 150‐mm transparent walls) and a centre area (W 50 mm, D 50 mm), for 10 min. The experimental room was illuminated at 100 lux. Animals were positioned in the centre area when the experiment was started and allowed to move freely during the test. The total distance travelled, time spent in each arm and the number of entries to arms were recorded.

#### 2.4.6 Rotarod test

The rotarod test was performed with a rotarod apparatus (Ugo Basile, Varese, Italy). Six trials (3 trials/day × 2 days) were conducted. The testing room was illuminated at 100 lux. Animals were positioned on a rotating rod (4 rpm, 30 mm in diameter) when the experiment was started. Speed of rotation was gradually accelerated from 4 to 40 rpm over 5 min. The latency to fall was recorded (max 300 s).

#### 2.4.7 Hot plate test

The hot plate test was performed with a hot plate apparatus (W 255 mm, D 255 mm; Columbus Instruments International., Ohio, USA). The experimental room was illuminated at 100 lux. Animals were positioned on a 55°C hot plate. Animals moved freely on the hot plate during the test. The latency to the first foot shake or paw lick was recorded (max 16 s).

#### 2.4.8 Crawley's social interaction test

The Crawley's social interaction test was performed in an arena consisted of three chambers (W 200 mm, D 400 mm, H 300 mm each; O'HARA, Tokyo, Japan). On the first day, all stranger mice were habituated to the small cages in the arena for 10 min. On the second day, subject animals were first placed in the arena with a stranger caged mouse in one side, for 10 min (mouse cage vs. empty cage). Subsequently, subject animals were removed and again placed in the same arena with the previous stranger mouse on the same side with a novel stranger mouse on another side, for 10 min (familiar mouse cage vs. novel mouse cage). See detailed methods in the supporting information.

#### 2.4.9 Porsolt swim test

The Porsolt swim test was performed in a small round pool (113 mm in diameter, H 216 mm; O'HARA, Tokyo, Japan). The pool was filled with 20°C hypochlorous acid water (pH 6.5 hypochlorous acid) to a height of 75 mm and placed in a white box (100 lux). The mice were tested on two consecutive days, 10 min/day. Animals were allowed to swim freely in the pool during the test. The percent of immobile time, that is, floating status, was recorded.

#### 2.4.10 Prepulse inhibition test

The prepulse inhibition test was performed in a startle reflex measurement box (O'HARA, Tokyo, Japan). Acoustic startle responses were measured by stimuli of 90, 100, 110, and 120 dB of white noise (40 ms, 1000–20,000 Hz). Subsequently, prepulse inhibition of acoustic startle responses was measured by pairs of 70 (pre)–120 dB, 75–120 dB, 80–120 dB and 85–120 dB of white noise (40 ms). See detailed methods in the supporting information.

#### 2.4.11 Barnes maze

The Barnes maze was performed on a white circular arena (1.0 m in diameter; O'HARA, Tokyo, Japan). Twelve holes (40 mm in diameter) were equally spaced around its circumference. The training session consisted of 16 trials (1 trial/day, 5 min). After 1 day and 8 days of training session, probe tests were conducted. See detailed methods in supporting information.

#### 2.4.12 Fear‐conditioning test

The fear‐conditioning test was performed over 3 days (apparatus were from O'HARA, Tokyo, Japan). On the first day, conditioning was conducted. On the second day, a contextual test was conducted. On the third day, a cue test was conducted. See detailed methods in the supporting information.

#### 2.4.13 Tail suspension test

The tail suspension test was performed for 10 min. The base of the tail of the mouse was taped onto a metal board, and animals were suspended 270 mm above the floor in a white box (100 lux; O'HARA, Tokyo, Japan). The percent of immobile time was recorded.

#### 2.4.14 Tail‐wound counting and ranking

The number of tail wounds (red or dark red scab, scratch and internal bleeding) from both ventral and dorsal sides was counted manually in the pre‐ST* and post‐ST* photos and added for each animal. The ER (EE ‐>ST*) animals with the fewest wounds in each post‐ST* cage were regarded as ‘ER_α’ animals (one α animal/cage) and the other two ‘ER_others’.

#### 2.4.15 Aggressive behaviour evaluation in ST* (video analysis)

The number of aggressive interactions in ST* was counted manually in recorded videos. Aggressive interactions include chasing, wrestling, boxing and mounting. When multiple aggressive interactions occurred within 3 s, they were regarded as a single continuous aggressive interaction thus counted as one (Sano et al., 2016). If multiple mounting behaviours occurred within 3 s, they were counted as one. A 60‐min video records starting 1 h after lights‐off were utilised for this analysis, based on the increased activity level and aggressive behaviour during the early dark phase (Todd et al., 2018).

#### 2.4.16 Activity level and social behaviour evaluation in ST* (video analysis)

Activity level data (the summation of the number of different pixels between consecutive two flames [8 flames/s] in each 1 min) and social behaviour data (the average of the number of particles; how many clusters of animals were in a cage in each 1 min) were recorded by the software and apparatus included in this system (O'HARA, Tokyo, Japan). If all three animals took distance from other animals, the number of particles was three. If all three animals stick together, the number of particles was one. For analysis, the average activity level data and social behaviour data in every 12 h were calculated by averaging each data of 1 min including each 12 h.

### 2.5 Statistical analysis

Statistical analysis and graphs were conducted using R version 4.0.0 (R Core Team, 2020). Analysis of variance (ANOVA) and Holm's sequentially rejective Bonferroni procedure (Holm's method) were carried out by R function “anovakun” version 4.8.5 (Iseki, 2020). For single‐factor experiments, we presented the outputs of Holm's method (e.g., bodyweight measurement). For two‐factor experiments, we presented the outputs of ANOVA and subsequent analysis by Holm's method (e.g., the prepulse inhibition test). We excluded animals from analysis when we noticed administrative failures of experimental procedures on them.

#### 2.5.1 SNP genotyping

DNA was extracted from the tail tissue with 200‐ul 50‐mM NaOH (FUJIFILM Wako Pure Chemical, Osaka, Japan) and amplified using Tks Gflex DNA Polymerase kit (Takara Bio, Shiga, Japan) on PCR Thermal Cycler Dice (Takara Bio). PCR products were analysed on 2% agarose gels (NIPPON GENE, Tokyo, Japan). Primers and the detailed procedure were according to Zhang et al. (2004). See methods in supporting information.

## 3 RESULTS

We summarised our results from Experiment 1 in Table 1 and from Experiment 2 in Tables 2 and 3.

**TABLE 1:** Summary of behavioural tests in Experiment 1

Experiment 1 behavioural test battery			
Test	Main index of the test	EE (compared with ST)	SI (compared with ST)
Body weight	Weight	↑	↑
Rectal temperature	Temperature	↑	↑
Grip strength	Strength	↑	↑
Wire hang	Latency to fall	‐‐	↓
Rotarod	Latency to fall	↑	‐‐
Hot plate	Latency to response	↓	‐‐
Acoustic startle response	Startle amplitude	↑	‐‐
Prepulse inhibition	Startle amplitude	↑	↑
Open‐field	Total distance travelled	↓	↑
	Time spent in the centre area	↑	↑
	Vertical activities	‐‐	↑
Light–dark box	Total distance travelled	‐‐	↑
	Light chamber staying	‐‐	↑
	Lateny to enter the light‐box	‐‐	‐‐
Elevated‐plus maze	Total distance travelled	↓	↑
	Open arms staying	‐‐	↑
	Number of open arm entries	‐‐	↑
Social interaction	Time spent with mice (vs. empty cage)	‐‐	‐‐
	Time spent with novel mice (vs. familiar mice)	‐‐	‐‐
Porsolt swim	Immobility	↑	↓
Tail suspension	Immobility	↑	↓
Y‐maze	Alternation	‐‐	‐‐
Fear‐conditioning	Freezing (context)	↑	↓
	Freezing (cue)	↑	↓
Barnes maze	Time spent around the target hole	(no data)	↓

Abbreviations: EE, enriched environment; SI, social isolation; ST, standard housing.

**TABLE 2:** Summary of video analysis and wound counts in ER in Experiment 2 compared with ST (from ST to different type of standard housing [ST*])

Experiment 2 video analysis and wound counting	
Index	ER
Tail wounds	↑
Chasing, wrestling, and boxing behaviour	↑
Mounting behaviour	↑
Activity level in the dark phase	↓
Activity level in the light phase	↑
Social distancing in the dark phase	↑
Social distancing in the light phase	↑

*Note*: ER: Enrichment removal (from EE to ST*).

**TABLE 3:** Summary of the behavioural test battery in Experiment 2

Experiment 2 behavioural test battery				
Test	Main index of the test	ER_α (compared with ST)	ER_other (compared with ST)	ER_α (compared with ER_other)
Open‐field	Total distance travelled	‐‐	↓	‐‐
	Time spent in the centre area	↑	↑	↓
	Vertical activities	↑	‐‐	↑
Social interaction	Time spent with mice (vs. empty cage)	‐‐	‐‐	‐‐
	Time spent with novel mice (vs. familiar mice)	‐‐	‐‐	‐‐
Tail suspension	Immobility	↑	↑	‐‐

*Note*: ER_α: alpha‐ranked animals of ER group. ER_other: lower‐ranked animals of ER group.

### 3.1 General health and motor function

#### 3.1.1 General health and neurological examination

General health and neurological examinations on whisker state, coat state, righting reflex, whisker twitch, ear twitch, reaching and epilepsy detected no abnormalities (data not shown).

In bodyweight (g) measurement (Figure 1b; 12 ST animals [*Mean* = 24.21, *SEM* = 0.31] vs. 10 EE animals [*Mean* = 27.74, *SEM* = 0.60] vs. 12 SI animals [*Mean* = 25.48, *SEM* = 0.32]), there were significant differences between ST and EE groups (*p* < .001, adjusted *p* < .001, *r* = .73), between ST and SI groups (*p* = .031, adjusted *p* = .031, *r* = .38) and between EE and SI groups (*p* = .001, adjusted *p* = .001, *r* = .57). EE animals had the heaviest body weight, and ST animals had the lightest weight.

In rectal temperature (°C) measurement (Figure 1c; 12 ST animals [*Mean* = 36.85, *SEM* = 0.16] vs. 10 EE animals [*Mean* = 37.36, *SEM* = 0.23] vs. 12 SI animals [*Mean* = 38.00, *SEM* = 0.12]), there were significant differences between ST and EE groups (*p* = .044, adjusted *p* = .044, *r* = .35), between ST and SI groups (*p* < .001, adjusted *p* < .001, *r* = .67) and between EE and SI groups (*p* = .012, adjusted *p* = .024, *r* = .43). SI animals had the highest, and ST animals had the lowest rectal temperature.

In grip strength measurement (Figure 1d; 12 ST animals vs. 10 EE animals vs. 12 SI animals), there were significant differences between ST and EE groups (*p* < .001, adjusted *p* < .001, *r* = .73) and between ST and SI groups (*p* < .001, adjusted *p* < .001, *r* = .66). EE and SI animals showed enhanced grip strength compared with ST animals.

The latency to fall in the wire hang test is shown in Figure 1e (12 ST animals vs. 10 EE animals vs. 12 SI animals). There were significant differences between ST and SI groups (*p* = .011, adjusted *p* = .021, *r* = .44) and between EE and SI groups (*p* < .001, adjusted *p* = .001, *r* = .60). SI animals dropped from the wire mesh earlier than ST and EE animals.

#### 3.1.2 Rotarod test

The latency to fall in day 1 is shown in Figure 1f left (12 ST animals vs. 8 EE animals vs. 12 SI animals). A 3 (housing; ST, EE and SI; between‐animal) × 3 (trial; within‐animal) ANOVA was conducted. The main effect of housing was significant [*F*(2, 29) = 9.78, *p* = .001, *η*_*p*_^*2*^ = .403]. The subsequent analysis revealed that there were significant differences between ST and EE groups (*p* < .001, adjusted *p* = .001, *r* = .62) and between EE and SI groups (*p* = .002, adjusted *p* = .003, *r* = .54). EE animals rode on rotarods longer than ST and SI animals in day 1. The main effect of trial was significant [*F*(2, 58) = 7.23, *p* = .002, *η*_*p*_^*2*^ = .200]. The interaction between housing and trial was not significant [*F*(4, 58) = 0.39, *p* = .818, *η*_*p*_^*2*^ = .026].

The latency to fall in day 2 is shown in Figure 1f right (12 ST animals vs. 8 EE animals vs. 12 SI animals). A 3 (housing; ST, EE, and SI; between‐animal) × 3 (trial; within‐animal) ANOVA was conducted. The main effect of housing was significant [*F*(2, 29) = 18.57, *p* < .001, *η*_*p*_^*2*^ = .562]. The subsequent analysis revealed that there were significant differences between ST and EE groups (*p* < .001, adjusted *p* < .001, *r* = .75) and between EE and SI groups (*p* < .001, adjusted *p* < .001, *r* = .63). EE animals stayed on the rotarod longer than ST and SI animals on day 2. The main effect of trial was significant [*F*(2, 58) = 7.64, *p* = .001, *η*_*p*_^*2*^ = .209]. The interaction between housing and trial was not significant [*F*(4, 58) = 0.31, *p* = .872, *η*_*p*_^*2*^ = .021].

The ratio of performances of day 2 trial 1/day 1 trial 3 is shown in Figure 1g (12 ST animals vs. 8 EE animals vs. 12 SI animals). There was no significant difference between ST and EE groups (*p* = .042, adjusted *p* = .126, *r* = .37), between ST and SI groups (*p* = .236, adjusted *p* = .472, *r* = .22) or between EE and SI groups (*p* = .304, adjusted *p* = .472, *r* = .19).

### 3.2 Sensory function

#### 3.2.1 Hot plate test

**FIGURE 2:**
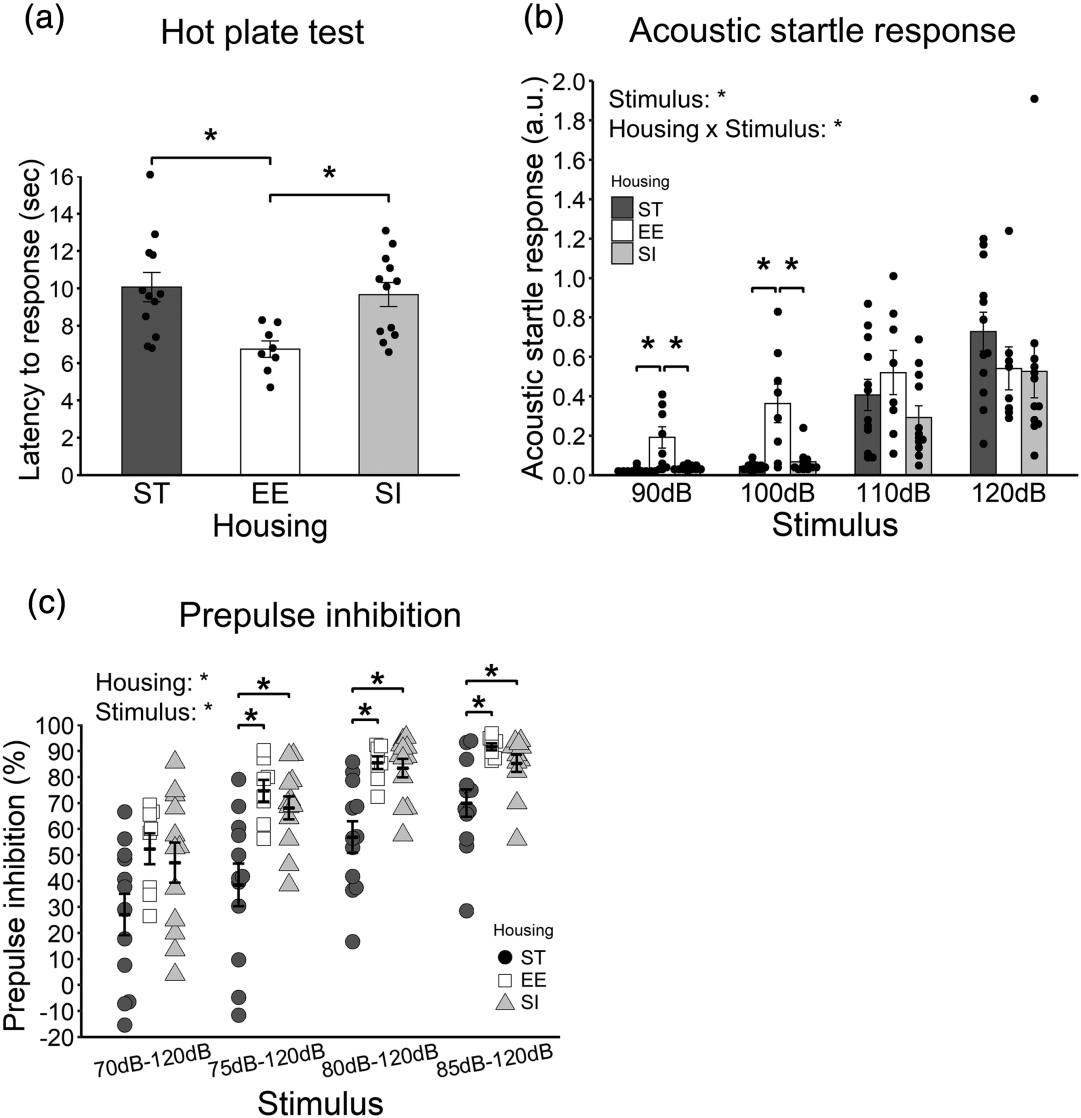
Sensory function. (a) Latency to response in the hot plate test. (b) Acoustic startle response. a.u.: arbitrary unit. (c) Prepulse inhibition. Bars in the centre represent the means. Error bars represent standard errors of the mean. Asterisks represent adjusted *p* < .05

The latency to response is shown in Figure 2a (12 ST animals vs. 8 EE animals vs. 12 SI animals). There were significant differences between ST and EE groups (*p* = .003, adjusted *p* = .009, *r* = .52) and between EE and SI groups (*p* = .008, adjusted *p* = .016, *r* = .47). EE animals responded earlier to foot heat than ST and SI animals.

#### 3.2.2 Prepulse inhibition test

Acoustic startle response is shown in Figure 2b (12 ST animals vs. 8 EE animals vs. 12 SI animals). A 3 (housing; ST, EE, and SI; between‐animal) × 4 (stimulus; within‐animal) ANOVA was conducted. The main effect of housing was not significant [*F*(2, 29) = 2.24, *p* = .125, *η*_*p*_^*2*^ = .134]. The main effect of stimulus was significant [*F*(3, 87) = 44.43, *p* < .001, *η*_*p*_^*2*^ = .605]. The interaction between housing and stimulus was significant [*F*(6, 87) = 3.00, *p* = .010, *η*_*p*_^*2*^ = .171]. The subsequent analysis revealed that there were significant differences at 90 dB between ST and EE groups (*p* < .001, adjusted *p* < .001, *r* = .66) and between EE and SI groups (*p* < .001, adjusted *p* < .001, *r* = .64), and there were significant differences at 100 dB between ST and EE groups (*p* < .001, adjusted *p* < .001, *r* = .68) and between EE and SI groups (*p* < .001, adjusted *p* < .001, *r* = .65). EE animals showed enhanced startle response compared with ST and SI animals to 90‐ and 100‐dB acoustic stimuli, indicating enhanced response to relatively small acoustic stimuli of EE animals.

Prepulse inhibition rate is shown in Figure 2c (12 ST animals vs. 8 EE animals vs. 12 SI animals). A 3 (housing; ST, EE, and SI; between‐animal) × 4 (stimulus; within‐animal) ANOVA was conducted. The main effect of housing was significant [*F*(2, 29) = 8.98, *p* = .001, *η*_*p*_^*2*^ = .383]. The subsequent analysis revealed that there were significant differences between ST and EE groups (*p* = .001, adjusted *p* = .002, *r* = .57) and between ST and SI groups (*p* = .002, adjusted *p* = .004, *r* = .54). EE and SI animals showed enhanced prepulse inhibition compared with ST animals. The main effect of stimulus was significant [*F*(3, 87) = 72.05, *p* < .001, *η*_*p*_^*2*^ = .713]. The interaction between housing and stimulus was not significant [*F*(6, 87) = 1.16, *p* = .334, *η*_*p*_^*2*^ = .074].

### 3.3 Activity level and anxiety‐like behaviour

#### 3.3.1 Open‐field test

**FIGURE 3:**
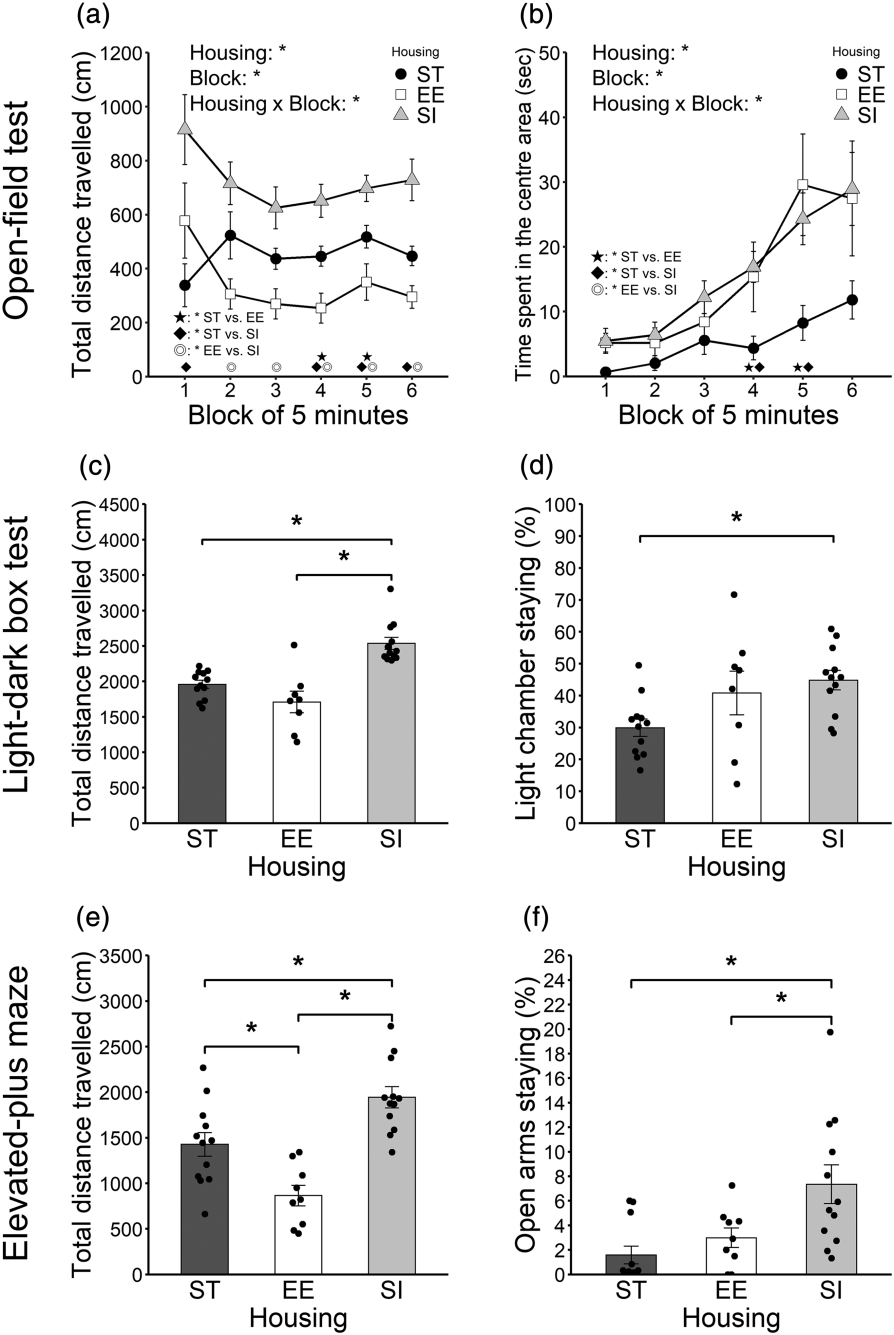
Activity level and anxiety‐like behaviour. (a) Total distance travelled in the open‐field test. (b) Time spent in the centre area in the open‐field test. (c) Total distance travelled in the light–dark box test. (d) Percent of time spent in the light chamber in the light–dark box test. (e) Total distance travelled in the elevated‐plus maze. (f) Percent of time staying in open arms in the elevated‐plus maze. Error bars represent standard errors of the mean. Asterisks represent adjusted *p* < .05

The total distance travelled is shown in Figure 3a (12 ST animals vs. 9 EE animals vs. 12 SI animals). A 3 (housing; ST, EE, and SI; between‐animal) × 6 (block; within‐animal) ANOVA was conducted. The main effect of housing was significant [*F*(2, 30) = 12.57, *p* < .001, *η*_*p*_^*2*^ = .456]. The subsequent analysis revealed that there were significant differences between ST and SI groups (*p* = .001, adjusted *p* = .002, *r* = .55) and between EE and SI groups (*p* < .001, adjusted *p* < .001, *r* = .65). SI animals showed longer total distance travelled than ST and EE animals. The main effect of block was significant [*F*(5, 150) = 3.93, *p* = .002, *η*_*p*_^*2*^ = .116]. The interaction between housing and block was significant [*F*(10, 150) = 3.39, *p* = .001, *η*_*p*_^*2*^ = .184]. The subsequent analysis revealed that EE animals travelled less than ST or SI animals at all but the first block, and SI animals travelled more than ST or EE animals at all blocks.

Time spent in the centre area is shown in Figure 3b (12 ST animals vs. 9 EE animals vs. 12 SI animals). A 3 (housing; ST, EE, and SI; between‐animal) × 6 (block; within‐animal) ANOVA was conducted. The main effect of housing was significant [*F*(2, 30) = 5.02, *p* = .013, *η*_*p*_^*2*^ = .251]. The subsequent analysis revealed that there were significant differences between ST and EE groups (*p* = .017, adjusted *p* = .034, *r* = .42) and between ST and SI groups (*p* = .008, adjusted *p* = .023, *r* = .46). EE and SI animals spent more time in the centre area than ST animal. The main effect of block was significant [*F*(5, 150) = 26.23, *p* < .001, *η*_*p*_^*2*^ = .467]. The interaction between housing and block was significant [*F*(10, 150) = 2.26, *p* = .017, *η*_*p*_^*2*^ = .131]. The subsequent analysis revealed that EE and SI animals spent more time in the centre area than ST animals at the fourth and fifth blocks.

The number of vertical activities is shown in Figure S2 (12 ST animals vs. 9 EE animals vs. 12 SI animals). A 3 (housing; ST, EE, and SI; between‐animal) × 6 (block; within‐animal) ANOVA was conducted. The main effect of housing was significant [*F*(2, 30) = 11.83, *p* < .001, *η*_*p*_^*2*^ = .441]. The subsequent analysis revealed that there were significant differences between ST and SI groups (*p* < .001, adjusted *p* < .001, *r* = .66) and between EE and SI groups (*p* = .004, adjusted *p* = .009, *r* = .49). SI animals showed more vertical activities than ST and EE animals. The main effect of block was significant [*F*(5, 150) = 15.32, *p* < .001, *η*_*p*_^*2*^ = .338]. The interaction between housing and block was not significant [*F*(10, 150) = 1.60, *p* = .112, *η*_*p*_^*2*^ = .096].

#### 3.3.2 Light–dark box test

The total distance travelled is shown in Figure 3c (12 ST animals vs. 8 EE animals vs. 12 SI animals). There were significant differences between ST and SI groups (*p* < .001, adjusted *p* < .001, *r* = .66) and between EE and SI groups (*p* < .001, adjusted *p* < .001, *r* = .74). SI animals travelled more than ST and EE animals.

The percent of time animals spent in the light chamber is shown in Figure 3d (12 ST animals vs. 8 EE animals vs. 12 SI animals). There were significant differences between ST and SI groups (*p* = .008, adjusted *p* = .025, *r* = .47). SI animals spent more time in the light chamber than ST animals.

The latency to enter the light chamber is shown in Figure S3 (12 ST animals vs. 8 EE animals vs. 12 SI animals). There was no significant difference between ST and EE groups (*p* = .093, adjusted *p* = .280, *r* = .31), between ST and SI groups (*p* = .692, adjusted *p* = .692, *r* = .07) or between EE and SI groups (*p* = .179, adjusted *p* = .358, *r* = .25).

#### 3.3.3 Elevated‐plus maze

The total distance travelled is shown in Figure 3e (12 ST animals vs. 9 EE animals vs. 12 SI animals). There were significant differences between ST and EE groups (*p* = .004, adjusted *p* = .008, *r* = .50), between ST and SI groups (*p* = .004, adjusted *p* = .008, *r* = .50) and EE and SI group (*p* < .001, adjusted *p* < .001, *r* = .74). SI animals showed the longest and EE animals showed the shortest total distance travelled.

The percent of time staying in open arms is shown in Figure 3f (12 ST animals vs. 9 EE animals vs. 12 SI animals). There were significant differences between ST and SI groups (*p* = .001, adjusted *p* = .003, *r* = .56) and between EE and SI groups (*p* = .015, adjusted *p* = .030, *r* = .43). SI animals spent more time in open arms than ST and EE animals.

The number of entries into arms is shown in Figure S4 (12 ST animals vs. 9 EE animals vs. 12 SI animals). A 3 (housing; ST, EE and SI; between‐animal) × 2 (arm; close and open; within‐animal) ANOVA was conducted. The main effect of housing was significant [*F*(2, 30) = 13.62, *p* < .001, *η*_*p*_^*2*^ = .476]. The subsequent analysis revealed that there were significant differences at closed arms between ST and EE groups (*p* = .033, adjusted *p* = .033, *r* = .38), between ST and SI groups (*p* = .004, adjusted *p* = .008, *r* = .50) and EE and SI groups (*p* < .001, adjusted *p* < .001, *r* = .68). SI animals entered to arms most frequently, and EE animals entered to arms least frequently. The main effect of arm was significant [*F*(1, 30) = 153.13, *p* < .001, *η*_*p*_^*2*^ = .836]. The interaction between housing and arm was significant [*F*(2, 30) = 10.35, *p* < .001, *η*_*p*_^*2*^ = .408]. The subsequent analysis revealed that SI animals entered to closed arms most frequently and EE animals entered to closed arms least frequently, and SI animals entered to open arms more than ST animals.

### 3.4 Sociality

#### 3.4.1 Crawley's social interaction test

**FIGURE 4:**
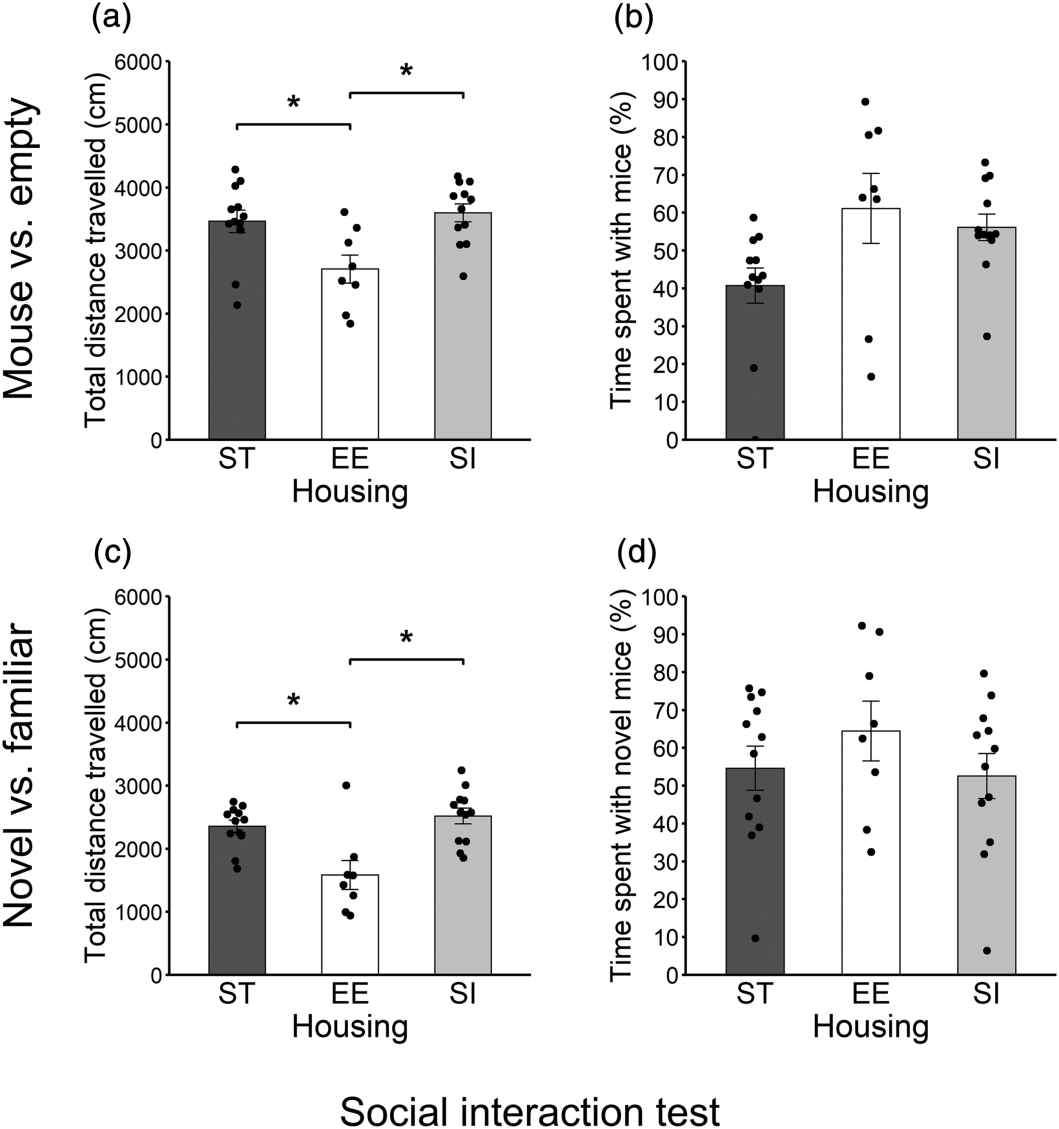
Sociality (Crawley's social interaction test). (a) Total distance travelled in the trial of mouse cage versus empty cage. (b) Percent of time staying around the mouse cage in the trial of mouse cage versus empty cage. (c) Total distance travelled in the trial of novel mouse cage versus familiar mouse cage. (d) Percent of time staying around the novel mouse cage in the trial of novel mouse cage versus familiar mouse cage. Error bars represent standard errors of the mean

The total distance travelled in the mouse cage versus empty cage trial is shown in Figure 4a (12 ST animals vs. 8 EE animals vs. 12 SI animals). There were significant differences between ST and EE groups (*p* = .008, adjusted *p* = .015, *r* = .47) and between EE and SI groups (*p* = .002, adjusted *p* = .006, *r* = .53). EE animals showed shorter total distance travelled than ST and SI animals.

The percent of time staying around the novel mouse cage is shown in Figure 4b (12 ST animals vs. 8 EE animals vs. 12 SI animals). There was no significant difference between ST and EE groups (*p* = .019, adjusted *p* = .056, *r* = .42), between ST and SI groups (*p* = .044, adjusted *p* = .088, *r* = .36) or between EE and SI groups (*p* = .545, adjusted *p* = .545, *r* = .11).

The total distance travelled in the familiar mouse cage versus novel mouse cage is shown in Figure 4c (12 ST animals vs. 8 EE animals vs. 12 SI animals). There were significant differences between ST and EE groups (*p* = .001, adjusted *p* = .002, *r* = .56) and between EE and SI groups (*p* < .001, adjusted *p* < .001, *r* = .63). EE animals showed shorter total distance travelled than ST and SI animals.

The percent of time staying around the novel mouse cage is shown in Figure 4d (12 ST animals vs. 8 EE animals vs. 12 SI animals). There was no significant difference between ST and EE groups (*p* = .312, adjusted *p* = .665, *r* = .19), between ST and SI groups (*p* = .807, adjusted *p* = .807, *r* = .05) or between EE and SI groups (*p* = .222, adjusted *p* = .665, *r* = .23).

### 3.5 Stress‐coping strategy

#### 3.5.1 Porsolt swim test

**FIGURE 5:**
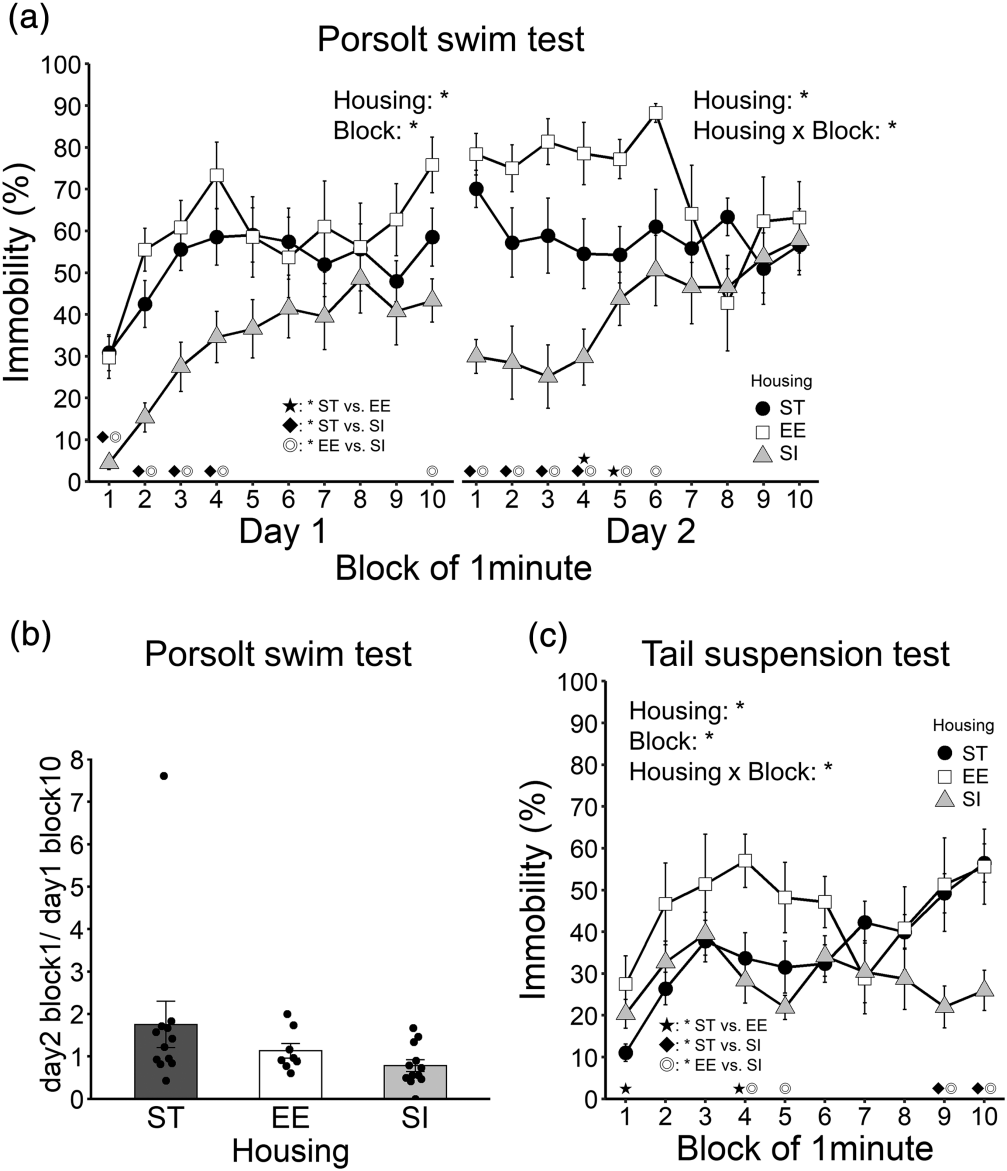
Stress‐coping strategy. (a) Percent of immobile time in the Porsolt swim test. (b) Ratio of immobile percent of day2 block 1/day1 block 10 in the Porsolt swim test. (c) Percent of immobile time in the tail suspension test. Error bars represent standard errors of the mean. Asterisks represent adjusted *p* < .05

The percent of immobile time in day 1 is shown in Figure 5a left (12 ST animals vs. 8 EE animals vs. 12 SI animals). A 3 (housing; ST, EE, and SI; between‐animal) × 10 (block; within‐animal) ANOVA was conducted. The main effect of housing was significant [*F*(2, 29) = 13.15, *p* < .001, *η*_*p*_^*2*^ = .476]. The subsequent analysis revealed that there were significant differences between ST and SI groups (*p* = .001, adjusted *p* = .001, *r* = .58) and between EE and SI groups (*p* < .001, adjusted *p* < .001, *r* = .66). SI animals showed lower immobility than ST and EE animals on day 1. The main effect of block was significant [*F*(9, 261) = 8.96, *p* < .001, *η*_*p*_^*2*^ = .236]. The interaction between housing and block was not significant [*F*(18, 261) = 1.03, *p* = .425, *η*_*p*_^*2*^ = .066].

The percent of immobile time on day 2 is shown in Figure 5a right (12 ST animals vs. 8 EE animals vs. 12 SI animals). A 3 (housing; ST, EE, and SI; between‐animal) × 10 (block; within‐animal) ANOVA was conducted. The main effect of housing was significant [*F*(2, 29) = 10.57, *p* < .001, *η*_*p*_^*2*^ = .422]. The subsequent analysis revealed that there were significant differences between ST and SI groups (*p* = .008, adjusted *p* = .015, *r* = .47) and between EE and SI groups (*p* < .001, adjusted *p* < .001, *r* = .64). Again, SI animals showed lower immobility than ST and EE animals on day 2. The main effect of block was not significant [*F*(9, 261) = 1.26, *p* = .258, *η*_*p*_^*2*^ = .042]. The interaction between housing and block was significant [*F*(18, 261) = 3.14, *p* < .001, *η*_*p*_^*2*^ = .178]. The subsequent analysis revealed that EE animals showed higher immobility than ST or SI animals in blocks 1–6, and SI animals showed lower immobility than ST or EE animals in blocks 1–6.

The ratio of the immobile percent of day 2 block 1/day 1 block 1 is shown in Figure 5b (12 ST animals vs. 8 EE animals vs. 12 SI animals). There was no significant difference between ST and EE groups (*p* = .278, adjusted *p* = .556, *r* = .20), between ST and SI groups (*p* = .063, adjusted *p* = .189, *r* = .34) or between EE and SI groups (*p* = .538, adjusted *p* = .556, *r* = .11).

#### 3.5.2 Tail suspension test

The percent of immobile time is shown in Figure 5c (12 ST animals vs. 7 EE animals vs. 12 SI animals). A 3 (housing; ST, EE, and SI; between‐animal) × 10 (block; within‐animal) ANOVA was conducted. The main effect of housing was significant [*F*(2, 28) = 5.23, *p* = .012, *η*_*p*_^*2*^ = .272]. The subsequent analysis revealed that there was a significant difference between EE and SI groups (*p* = .003, adjusted *p* = .010, *r* = .52). SI animals showed lower immobility than EE animals. The main effect of block was significant [*F*(9, 252) = 5.67, *p* < .001, *η*_*p*_^*2*^ = .168]. The interaction between housing and block was significant [*F*(18, 252) = 2.87, *p* < .001, *η*_*p*_^*2*^ = .170]. The subsequent analysis revealed that EE animals showed higher immobility than ST or SI animals in blocks 1, 4, 5, 9 and 10, and SI animals showed lower immobility than ST or EE animals in blocks 4, 5, 9 and 10.

### 3.6 Spatial working memory and associative fear memory

#### 3.6.1 Y‐maze

**FIGURE 6:**
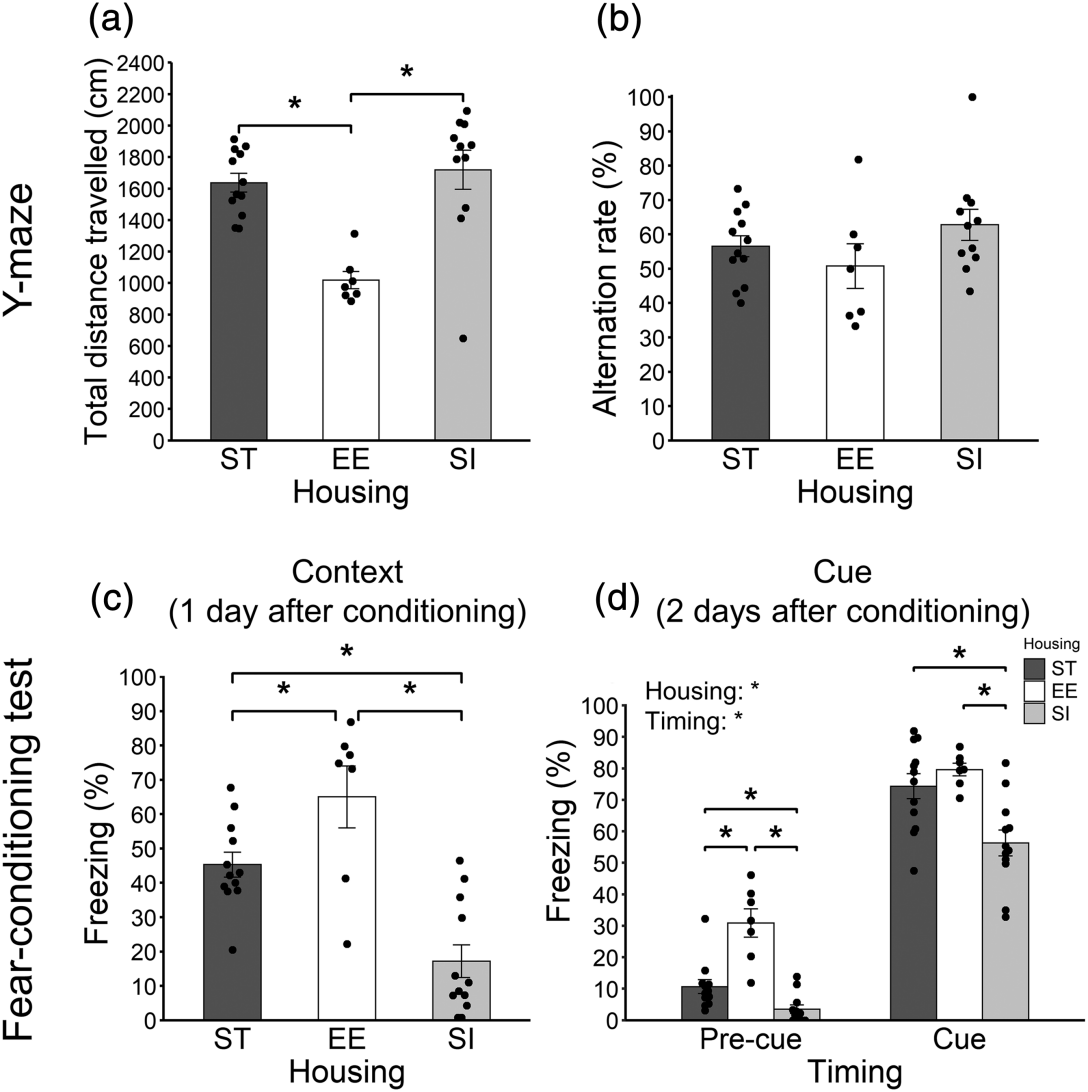
Spatial working memory and associative fear memory. (a) Total distance travelled in the Y‐maze. (b) Alternation rate in the Y‐maze. (c) Percent of freezing time in contextual memory test. (d) Percent of freezing time in cued memory test. Error bars represent standard errors of the mean. Asterisks represent adjusted *p* < .05

The total distance travelled is shown in Figure 6a (12 ST animals vs. 7 EE animals vs. 11 SI animals). There were significant differences between ST and EE groups (*p* < .001, adjusted *p* < .001, *r* = .65) and between EE and SI groups (*p* < .001, adjusted *p* < .001, *r* = .69). EE animals showed shorter total distance travelled than ST and SI animals.

Alternation rate is shown in Figure 6b (12 ST animals vs. 7 EE animals vs. 11 SI animals). There was no significant difference between ST and EE groups (*p* = .390, adjusted *p* = .590, *r* = .17), between ST and SI groups (*p* = .295, adjusted *p* = .590, *r* = .20) or between EE and SI groups (*p* = .086, adjusted *p* = .259, *r* = .32).

#### 3.6.2 Fear‐conditioning test

Percent of freezing time in context test is shown in Figure 6c (12 ST animals vs. 7 EE animals vs. 12 SI animals). There were significant differences between ST and EE groups (*p* = .022, adjusted *p* = .022, *r* = .42), between ST and SI groups (*p* < .001, adjusted *p* = .001, *r* = .61) and between EE and SI groups (*p* < .001, adjusted *p* < .001, *r* = .74). EE animals showed most, and SI animals showed least, freezing.

Percent of freezing time in the cue test is shown in Figure 6d (12 ST animals vs. 7 EE animals vs. 12 SI animals). A 3 (housing; ST, EE and SI; between‐animal) × 2 (timing; within‐animal) ANOVA was conducted. The main effect of housing was significant [*F*(2, 28) = 22.97, *p* < .001, *η*_*p*_^*2*^ = .621]. The subsequent analysis revealed that there were significant differences between ST and EE groups (*p* = .002, adjusted *p* = .002, *r* = .54), between ST and SI groups (*p* = .001, adjusted *p* = .001, *r* = .59) and between EE and SI groups (*p* < .001, adjusted *p* < .001, *r* = .78). EE animals showed most, and SI animals showed least, freezing. The main effect of timing was significant [*F*(1, 28) = 460.46, *p* < .001, *η*_*p*_^*2*^ = .943]. The interaction between housing and timing was not significant [*F*(2, 28) = 3.12, *p* = .060, *η*_*p*_^*2*^ = .182].

### 3.7 Spatial reference memory

#### 3.7.1 Barnes maze: Training session

In the training session, EE mice did not escape to holes, suggesting less motivation to avoid bright light and a spacious open place. We excluded EE animals from this test at training trial 12, because it is likely the result could not be used to appropriately evaluate the learning and memory functions of EE animals. The results of the training session are shown in Figure S5.

#### 3.7.2 Barnes maze: Probe tests

**FIGURE 7:**
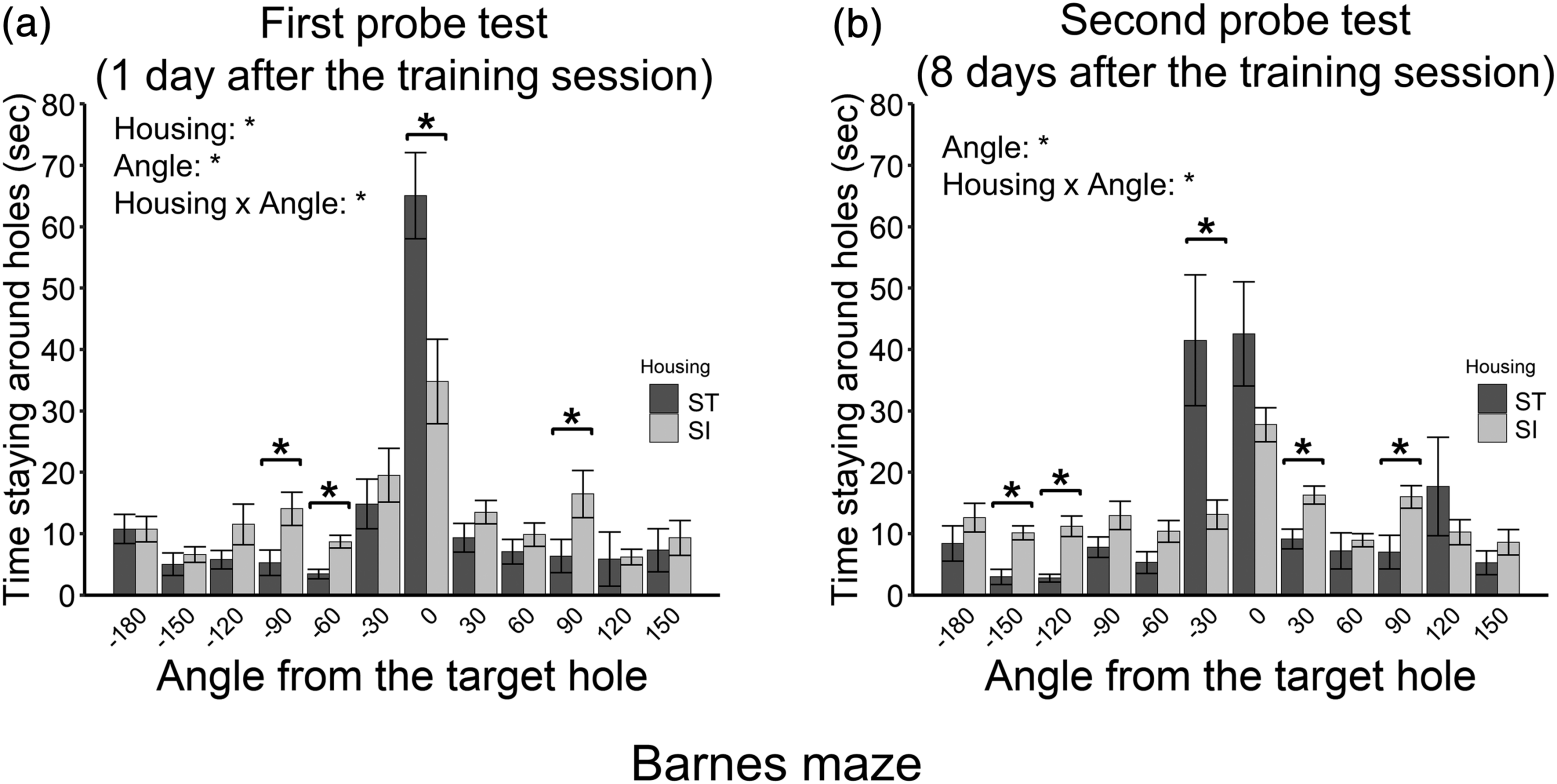
Spatial reference memory (the Barnes maze). (a) Time staying around holes in the first probe test. (b) Time staying around holes in the second probe test. Error bars represent standard errors of the mean. Asterisks represent adjusted *p* < .05

Time staying around holes in the first probe test (1‐day post‐training) is shown in Figure 7a (12 ST animals vs. 12 SI animals). A 2 (housing; ST and SI; between‐animal) × 12 (angle; within‐animal) ANOVA was conducted. The main effect of housing was significant [*F*(1, 22) = 7.86, *p* = .010, *η*_*p*_^*2*^ = .263], indicating that SI animals stayed around the area of holes longer than ST animals. The main effect of angle was significant [*F*(11, 242) = 25.67, *p* < .001, *η*_*p*_^*2*^ = .539]. The interaction between housing and angle was significant [*F*(11, 242) = 4.74, *p* < .001, *η*_*p*_^*2*^ = .177]. The subsequent analysis revealed that ST animals stayed around the target hole (0 angles) longer than SI animals, whereas SI animals stayed around holes of −90, −60 and 90 angles longer than ST animals.

Time staying around holes in the second probe test (eight days post‐training) is shown in Figure 7b (12 ST animals vs. 12 SI animals). A 2 (housing; ST and SI; between‐animal) × 12 (angle; within‐animal) ANOVA was conducted. The main effect of housing was not significant [*F*(1, 22) = 0.02, *p* = .897, *η*_*p*_^*2*^ < .001], indicating that SI animals stayed around the area of holes longer than ST animals. The main effect of angle was significant [*F*(11, 242) = 10.55, *p* < .001, *η*_*p*_^*2*^ = .324]. The interaction between housing and angle was significant [*F*(11, 242) = 4.26, *p* < .001, *η*_*p*_^*2*^ = .162]. The subsequent analysis revealed that ST animals stayed around the hole of 30 angles longer than SI animals, whereas SI animals stayed around holes of −150, −120, 30 and 90 angles longer than ST animals.

### 3.8 Fighting behaviour under ER

#### 3.8.1 Tail wounds counting in pre‐ST* and post‐ST*

**FIGURE 8:**
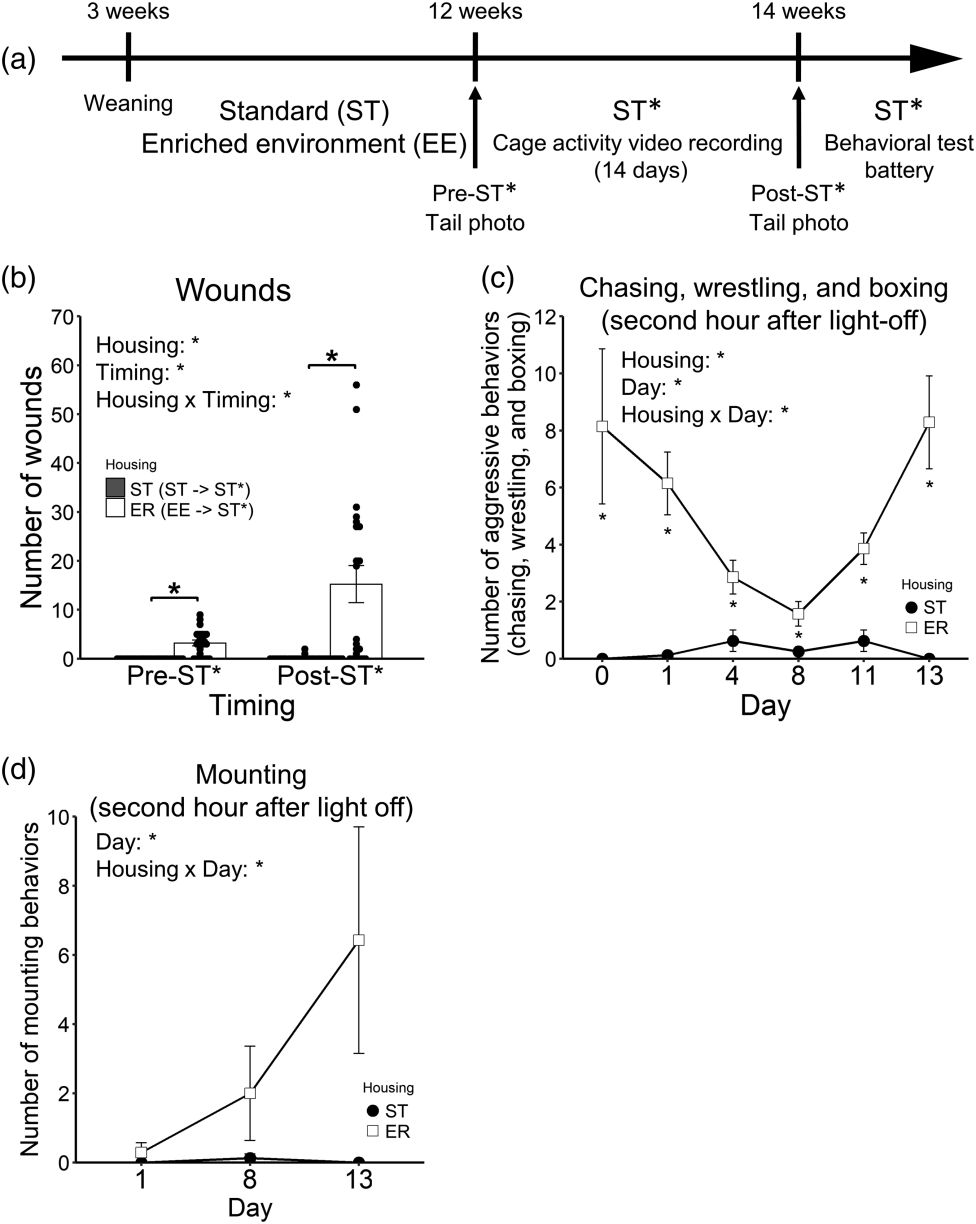
Fighting behaviour under enrichment removal. (a) The schema of Experiment 2. (b) the number of tail wounds pre‐ST* and post‐ST*. (c) The number of aggressive interactions (chasing, wrestling, and boxing) within the second hour after light‐off in ST*. (d) The number of mounting behaviour within the second hour after light‐off in ST*. Asterisks represent adjusted *p* < .05

The numbers of tail wounds before and after ST* are shown in Figure 8b (24 ST [ST ‐ > ST*] animals vs. 21 ER [EE ‐ > ST*] animals). A 2 (housing; ST and ER; between‐animal) × 2 (timing; pre‐ST* and post‐ ST*; within‐animal) ANOVA was conducted. The main effect of housing was significant [*F*(1, 43) = 22.98, *p* < .001, *η*_*p*_^*2*^ = .348], indicating that ER animals had more wounds than ST animals. The main effect of timing was significant [*F*(1, 43) = 12.63, *p* = .001, *η*_*p*_^*2*^ = .227]. The interaction between housing and timing was significant [*F*(1, 43) = 12.81, *p* = .001, *η*_*p*_^*2*^ = .230]. The subsequent analysis revealed that ER animals had more wounds than ST animals in both pre‐ST* and post‐ST*; furthermore, the number of wounds of ER animals increased in post‐ST* compared with pre‐ST*.

#### 3.8.2 The number of aggressive behaviour (chasing, wrestling and boxing) under ST*(video analysis)

The numbers of aggressive behaviour (chasing, wrestling, and boxing) under ST* are shown in Figure 8c (eight ST cages vs.seven ER cages). A 2 (housing; ST and ER; between‐animal) × 6 (day; within‐animal) ANOVA was conducted. The main effect of housing was significant [*F*(1, 13) = 88.10, *p* < .001, *η*_*p*_^*2*^ = .871], indicating that ER animals showed chasing, wrestling and boxing more often than ST animals. The main effect of day was significant [*F*(5, 65) = 3.67, *p* = .006, *η*_*p*_^*2*^ = .220]. The interaction between housing and day was significant [*F*(5, 65) = 4.97, *p* = .001, *η*_*p*_^*2*^ = .277], indicating different temporal dynamics of aggressive behaviour between ST and ER animals. The subsequent analysis did not show any significant differences between any days on the data of ER animals.

#### 3.8.3 The number of mounting behaviour under ST* (video analysis)

The number of mounting behaviour under ST* is shown in Figure 8d (eight ST cages vs. seven ER cages). A 2 (housing; ST and ER; between‐animal) × 3 (day; within‐animal) ANOVA was conducted. The main effect of housing was not significant [*F*(1, 13) = 3.81, *p* = .073, *η*_*p*_^*2*^ = .223]. The main effect of day was significant [*F*(2, 26) = 4.41, *p* = .022, *η*_*p*_^*2*^ = .254]. The interaction between housing and day was significant [*F*(2, 26) = 4.51, *p* = .021, *η*_*p*_^*2*^ = .258], indicating different temporal dynamics of mounting behaviour between ST and ER animals. The subsequent analysis did not show any significant differences between any days on the data of ER animals.

### 3.9 Activity level and social behaviour under ER

#### 3.9.1 Activity level

**FIGURE 9:**
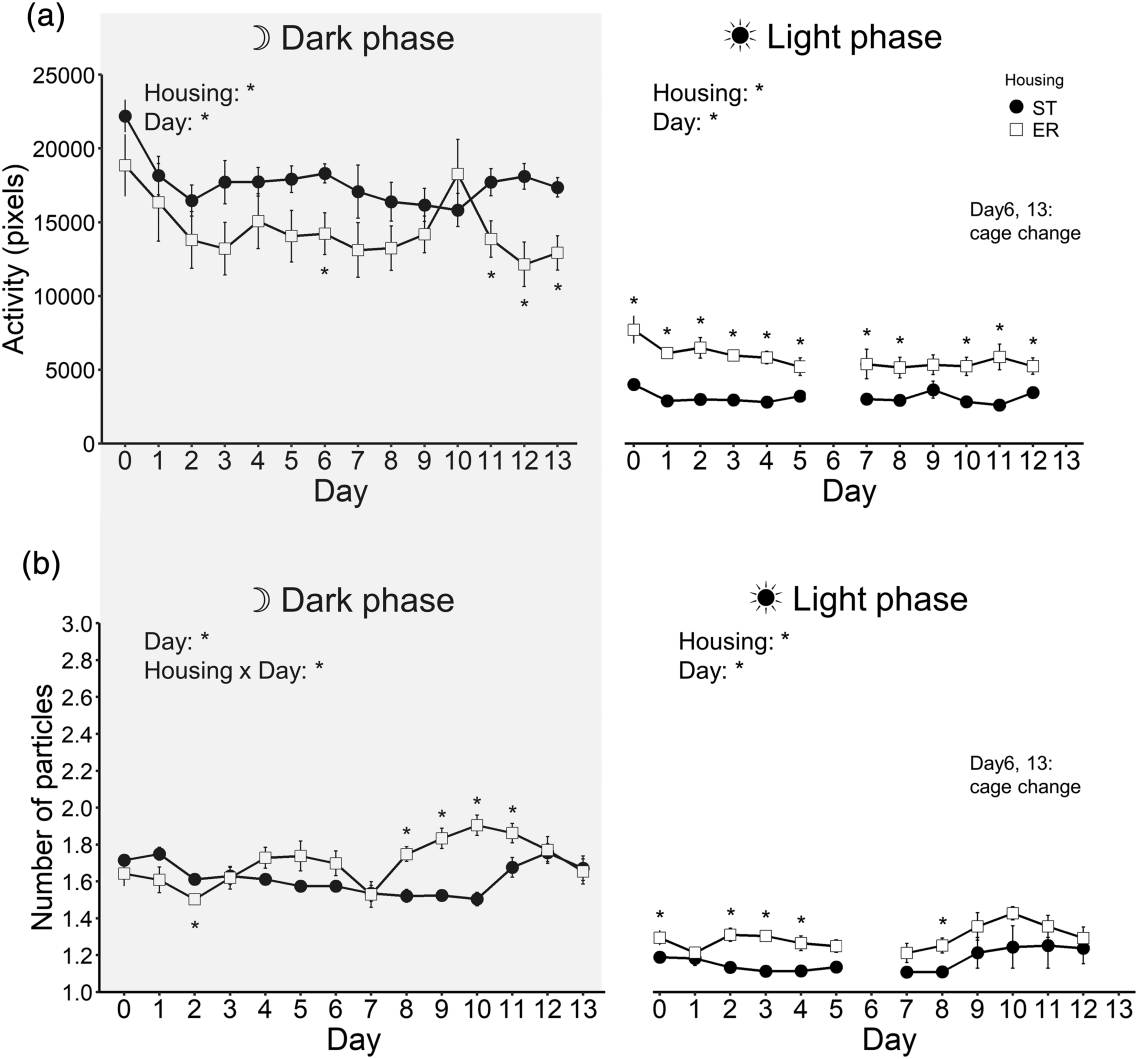
Activity level and social behaviour under enrichment removal. (a) Average of the activity level in the dark phase (left) and the light phase (right). (b) The average of number of particles of animals in the dark phase (left) and the light phase (right). Error bars represent standard errors of the mean. Asterisks represent adjusted *p* < .05

The average of activity level in the dark phase is shown in Figure 9a left (eight ST cages vs. seven ER cages). A 2 (housing; ST and ER; between‐animal) × 14 (day; within‐animal) ANOVA was conducted. The main effect of housing was significant [*F*(1, 13) = 5.73, *p* = .033, *η*_*p*_^*2*^ = .306], indicating that ER animals showed lower activity level than ST animals in the dark phase. The main effect of day was significant [*F*(13, 169) = 3.27, *p* < .001, *η*_*p*_^*2*^ = .201]. The interaction between housing and day was not significant [*F*(13, 169) = 1.39, *p* = .170, *η*_*p*_^*2*^ = .096].

The average of activity level in the light phase is shown in Figure 9a right (eight ST cages vs. seven ER cages). A 2 (housing; ST and ER; between‐animal) × 12 (day; within‐animal) ANOVA was conducted. The main effect of housing was significant [*F*(1, 13) = 28.75, *p* < .001, *η*_*p*_^*2*^ = .689], indicating that ER animals showed higher activity level than ST animals in the light phase. The main effect of day was significant [*F*(11, 143) = 3.23, *p* = .001, *η*_*p*_^*2*^ = .199]. The interaction between housing and day was not significant [*F*(11, 143) = 1.62, *p* = .099, *η*_*p*_^*2*^ = .111].

#### 3.9.2 Social behaviour

The average of number of particles in the dark phase is shown in Figure 9b left (eight ST cages vs.seven ER cages). A 2 (housing; ST and ER; between‐animal) × 14 (day; within‐animal) ANOVA was conducted. The main effect of housing was not significant [*F*(1, 13) = 3.87, *p* = .071, *η*_*p*_^*2*^ = .230]. The main effect of day was significant [*F*(13, 169) = 4.93, *p* < .001, *η*_*p*_^*2*^ = .275]. The interaction between housing and day was significant [*F*(13, 169) = 7.51, *p* < .001, *η*_*p*_^*2*^ = .366]. The subsequent analysis revealed that ER animals kept longer distances from other cage mates than ST animals in the dark phase on days 8, 9, 10 and 11, whereas ST animals kept longer distances than ER animals on day 2.

The average of number of particles in the light phase is shown in Figure 9b right (eight ST cages vs.seven ER cages). A 2 (housing; ST and ER; between‐animal) × 12 (day; within‐animal) ANOVA was conducted. The main effect of housing was significant [*F*(1, 13) = 5.14, *p* = .041, *η*_*p*_^*2*^ = .283], indicating that ER animals are more distanced from other cage mates than ST animals in the light phase. The main effect of day was significant [*F*(11, 143) = 1.97, *p* = .035, *η*_*p*_^*2*^ = .132]. The interaction between housing and day was not significant [*F*(11, 143) = 0.63, *p* = .804, *η*_*p*_^*2*^ = .046].

### 3.10 Activity level and anxiety‐like behaviour of ER animals

The average number of wounds in ER_α animals after ST* was 0.14 (seven animals, *SEM* = 0.14) and in ER_other animals was 22.79 (14 animals, *SEM* = 4.51).

#### 3.10.1 Open‐field test

The total distance travelled is shown in Figure S6 (24 ST animals vs. 7 ER_α animals vs. 14 ER_other animals). A 3 (group; ST, ER_α, and ER_other; between‐animal) × 6 (block; within‐animal) ANOVA was conducted. The main effect of group was significant [*F*(2, 42) = 4.74, *p* = .014, *η*_*p*_^*2*^ = .184]. The subsequent analysis revealed that there was significant difference between ST and ER_other groups (*p* = .004, adjusted *p* = .011, *r* = .43). ER_other animals showed less total distance travelled compared with ST animals. The main effect of block was significant [*F*(5, 210) = 60.02, *p* < .001, *η*_*p*_^*2*^ = .588]. The interaction between group and block was not significant [*F*(10, 210) = 1.17, *p* = .315, *η*_*p*_^*2*^ = .053].

Time spent in the centre area is shown in Figure S6 (24 ST animals vs. 7 ER_α animals vs. 14 ER_other animals). A 3 (group; ST, ER_α, and ER_other; between‐animal) × 6 (block; within‐animal) ANOVA was conducted. The main effect of group was significant [*F*(2, 42) = 9.22, *p* = .001, *η*_*p*_^*2*^ = .305]. The subsequent analysis revealed that there was a significant difference between ST and ER_other groups (*p* < .001, adjusted *p* < .001, *r* = .55). ER_other animals spent more time in the centre area than ST animals. The main effect of block was significant [*F*(5, 210) = 37.39, *p* < .001, *η*_*p*_^*2*^ = .471]. The interaction between group and block was significant [*F*(10, 210) = 4.04, *p* < .001, *η*_*p*_^*2*^ = .161]. The subsequent analysis revealed that ER_α animals spent more time in the centre area than ST animals at the sixth block, and ER_other animals spent more time in the centre area than ST animals or ER_α animals at all but the first block.

The numbers of vertical activities are shown in Figure S6 (24 ST animals vs. 7 ER_α animals vs. 14 ER_other animals). A 3 (group; ST, ER_α, and ER_other; between‐animal) × 6 (block; within‐animal) ANOVA was conducted. The main effect of group was significant [*F*(2, 42) = 7.00, *p* = .002, *η*_*p*_^*2*^ = .250]. The subsequent analysis revealed that there was a significant difference between ST and ER_α groups (*p* = .001, adjusted *p* = .003, *r* = .48). ER_α animals showed more vertical activities than ST animals. The main effect of block was significant [*F*(5, 210) = 25.80, *p* < .001, *η*_*p*_^*2*^ = 381]. The interaction between group and block was significant [*F*(10, 210) = 4.01, *p* < .001, *η*_*p*_^*2*^ = .160]. The subsequent analysis revealed that ER_α animals showed more vertical activities than ST animals or ER_other animals at all but the first two blocks.

### 3.11 The sociability of ER animals

#### 3.11.1 Crawley's social interaction test

The total distance travelled in the mouse cage versus empty cage trial is shown in Figure S7 (24 ST animals vs. 7 ER_α animals vs. 14 ER_other animals). There were significant differences between ST and ER_α groups (*p* = .001, adjusted *p* = .002 *r* = .49), between ST and ER_other groups (*p* < .001, adjusted *p* < .001, *r* = .77) and between ER_α and ER_other groups (*p* = .024, adjusted *p* = .024, *r* = .34). ST animals showed longest, and ER_other animals showed shortest total distance travelled.

The percent of time staying around the novel mouse cage is shown in Figure S7 (24 ST animals vs. 7 ER_α animals vs. 14 ER_other animals). There was no significant difference between ST and ER_α groups (*p* = .915, adjusted *p* = .915, *r* = .02), between ST and ER_other groups (*p* = .025, adjusted *p* = .076, *r* = .34) or between ER_α and ER_other groups (*p* = .120, adjusted *p* = .240, *r* = .24).

The total distance travelled in the familiar mouse cage versus novel mouse cage trial is shown in Figure S7 (24 ST animals vs. 7 ER_α animals vs. 14 ER_other animals). There were significant differences between ST and ER_α groups (*p* < .001, adjusted *p* < .001, *r* = .64) and between ST and ER_other groups (*p* < .001, adjusted *p* < .001, *r* = .72). ST animals showed longer total distance travelled than ER_α and ER_other animals.

The percent of time staying around the novel mouse cage is shown in Figure S7 (24 ST animals vs. 7 ER_α animals vs. 14 ER_other animals). There was no significant difference between ST and ER_α groups (*p* = .116, adjusted *p* = .348, *r* = .24), between ST and ER_other groups (*p* = .118, adjusted *p* = .348, *r* = .24) or between ER_α and ER_other groups (*p* = .745, adjusted *p* = .745, *r* = .05).

### 3.12 Stress‐coping strategy of ER animals

#### 3.12.1 Tail suspension test

The percent of immobile time is shown in Figure S8 (23 ST animals vs. 7 EE animals vs. 13 SI animals). A 3 (group; ST, ER_α, and ER_other; between‐animal) × 10 (block; within‐animal) ANOVA was conducted. The main effect of group was significant [*F*(2, 40) = 7.37, *p* = .002, *η*_*p*_^*2*^ = .269]. The subsequent analysis revealed that there was a significant difference between ST and ER_α groups (*p* = .001, adjusted *p* = .003, *r* = .49) and between ST and ER_other groups (*p* = .020, adjusted *p* = .040, *r* = .36). ST animals showed lower immobility than ER_α and ER_other animals. The main effect of block was significant [*F*(9, 360) = 9.23, *p* < .001, *η*_*p*_^*2*^ = .187]. The interaction between group and block was not significant [*F*(18, 360) = 1.29, *p* = .190, *η*_*p*_^*2*^ = .061].

### 3.13 SNP genotyping

To confirm a genetic SNP (C1473G) carried by tryptophan hydroxylase 2 (*Tph2*) gene in our animals, we PCR‐genotyped four BALB/cCrSIc mice and one C57BL/6 mouse. C57BL/6 mice were homozygous for 1473C, consistently with previous reports (Osipova et al., 2009). BALB/cCrSIc mice in this study were homozygous for 1473G (Figure S9), consistently with BALB/c mice in previous studies, indicating lower serotonin synthesis rate thus abnormal serotonin signalling in these animals.

## 4 DISCUSSION

In this study, we investigated the housing impacts of EE, SI and ER using male BALB/c mice. EE and SI housing affected the physiological states of animals and behavioural performance in various tests; ER altered social behaviour and daily/nightly activity levels.

Results from Experiment 1 indicate that long‐lasting manipulation of social and physical components of housing, EE and SI, can influence animal behaviour through multilayered processes. EE and SI altered lower‐level functions including fundamental physiological functions and motivation, which could have affected their task performance in higher‐function tests such as learning and memory. EE may have expanded the range of perspectives to the outer world and thus did not overreact (showed hypoactivity) to the transient novel environments during the tasks, while they had enhanced sensory and motor function. By contrast, SI housing deprives animals of sensory stimuli and social contacts. Possibly because of this deprivation, SI animals reacted to their outer world excessively (showed hyperactivity) during the tasks.

Our EE animals showed enhanced sensitivity to sensory input and enhanced prepulse inhibition. Regarding the effect of EE on sensory function, a limited number of previous studies reported inconsistent results among different strains (Abramov et al., 2008; Chen et al., 2010; Rabadán et al., 2019; Varty et al., 2000). The present study provides further insights into the impact of environmental factors on animal physiology and behaviour.

Behaviour of EE animals against the unavoidable stressors in the Porsolt swim test and the tail suspension test can be interpreted as more energy‐saving according to Commons et al. (2017). The results of day 2 of the Porsolt swim test could strongly reflect EE animals' cognitive enhancement. In previous reports, EE had ‘anti‐depressant like’ effect on the performance in the Porsolt swim test as, or no effect on it (Bogdanova et al., 2013). This difference could be originated from experimental conditions, and we could not exclude possibilities of influence of stress in our EE animals. Alternatively, this result could simply be related to the lower locomotor activity of EE animals during behavioural tests. About sociality, in Crawley's social interaction test, there was no significant difference between ST animals and EE animals. However, when comparing ST and EE data of the percent of time staying around the mouse cage, the effect size *r* is .42, not a very small value.

In the learning and memory task, the above behavioural patterns of the EE animals possibly influence their performance. EE animals were less active in the Y‐maze chamber. EE animals showed increased freezing in the fear‐conditioning test, which utilised foot shocks. These results may be related to their activity level in the test chamber and/or enhanced sensory function, as well as their learning and memory function.

SI animals showed contradictory results in the grip strength and wire hang tests. These results could be considered as evidence for their altered motivation in these tasks. In the sensory domain, SI animals showed enhanced prepulse inhibition, like stressed animals under chronic restraint (Chen et al., 2010). However, Geyer et al. (1993), Varty et al. (1995) and Wilkinson et al. (1994) found that SI animals had disrupted prepulse inhibition.

The hyperactivity of SI mice is consistent with many previous results (Walker et al., 2019). In contrast, SI has previously been shown in the Porsolt swim test as ‘depressogenic’ or ‘has no effect’ (Bogdanova et al., 2013). The differences could be possibly attributed to the different strains and/or animal ages during housing manipulation (Huang et al., 2021; Mesa‐Gresa et al., 2021).

In the learning and memory task, the above behavioural patterns of the SI animals possibly affect their performance. SI animals showed less freezing than ST animals in the contextual memory test and less staying time around the target or the next hole than ST animals in probe tests of the Barnes maze, despite their similar learning curve in the training session. Their baseline locomotor activity was higher, which could have affected task performance on testing memory function.

Although we could not exclude the effects of semi‐EE housing and animal fights during the test battery and the influence of experimental schedule in behavioural test battery, overall results regarding the effect of EE and SI were consistent with previous studies, except for the Porsolt swim test and the tail suspension test.

In Experiment 2, ER animals showed increased aggressive behaviour toward cage mates after being moved to ST*. The quantity and quality of aggressive behaviour such as chasing, wrestling, boxing and mounting continued to change. The temporal dynamics can be interpreted as follows: on day 0 or day 1 to exposure to ST*, the animals reacted to the abrupt environmental change to ST* with increased aggressive behaviour. On day 8, the animals were acclimated to ST* and showed less aggressive behaviour. On day 13, the combination of the establishment of new social relationships and the continuing burden of enrichment deprivation for 2 weeks increased aggressive behaviour again. ER animals were more socially distanced than ST animals in both dark and light phase. As previously reviewed by Gubert and Hannan (2019) and observed in our pre‐ST* wound counting experiment, EE itself could induce aggressive behaviour. However, how EE modulates social plasticity is largely unknown. Furthermore, the ER animals showed unorthodox activity levels in the dark and light phase, which could be related to stress‐induced insufficient rest.

In Experiment 2 behavioural test battery, ER animals travelled less distance and spent more time in the centre than ST mice in the open‐field test, which is reflective of less anxiety‐like behaviour. Especially, ER_α animals showed increased vertical movement which could be interpreted as enhanced environmental novelty‐seeking behaviour related to escape (C. Lever et al., 2006). In contrast, ER animals conserved their energy more than ST animals in the tail suspension test, according to the interpretation of Commons et al. (2017). Results from Experiment 2 were overall similar to results from Experiment 1.

Our results suggest that losing environmental privilege did not deprive ER animals of all previously obtained behavioural qualities in EE but induced acute and intense behavioural reactions to the ER situation, such as increased fighting, increased social distancing and unorthodox daily/nightly activity. The direct and detailed comparisons should be considered in future studies to evaluate and quantify the behavioural impact. For example, the altered total distance travelled in test chambers may possibly be because of the light exposure from the top of ST* cages for video recording in Experiment 2, which may have habituation effect on the animals in the test chambers.

Regarding to the genetic background of BALB/c mice, the homozygous 1473G SNP in the Tph2 gene in BALB/c mice could have affected behaviour via their lower activity in serotonin synthesis, thus generating serotonergic vulnerability (Osipova et al., 2009). This perspective of ER may help advance our understanding of the ‘loss’ mechanisms in analogy with drug (or other rewards) withdrawal after addiction. Moreover, the perspective of exclusive social behaviours (aggression and social distancing) and social hierarchy under ER is relevant to our societal issues, for example, increased domestic violence under behavioural restrictions due to COVID‐19 (Usher et al., 2020).

Limitations of our study include but are not restricted to the lacking of consideration of sex‐specific differences and detailed influences by social hierarchy in animal groups.

## 5 CONCLUSIONS

This study reveals that housing environment manipulation (EE, SI and ER) on the behaviour of BALB/c male mice could impact animal behaviour through multiple processes over a wide range of functional domains. The findings are relevant to understanding how environmental factors and altered sensory, cognitive and motor stimulation impact behaviour. Furthermore, the negative impact of stressors, such as SI or ER, is of relevance to understanding pathogenesis and developing novel therapeutic approaches, for a variety of brain disorders.

## AUTHOR CONTRIBUTIONS

MS, TY and DOW conceived and designed this project. MS performed all behavioural experiments and analyses. SH performed genotyping and analyses. TY and DOW supervised all experiments and analysis. MS, TY, MA, AJH and DOW wrote the manuscript.

## CONFLICT OF INTEREST

We have no conflicts of interest to disclose.

### PEER REVIEW

The peer review history for this article is available at https://publons.com/publon/10.1111/ejn.15602.

## Supporting information


**Figure S1.** EE with an open‐top arena and semi‐EE. (a) Light phase of EE. (b) Dark phase of EE. (c ) Semi‐EE where EE animals were kept during the behavioural test battery.
**Figure S2.** The number of vertical activities in the open‐field test. Error bars represent standard errors of the mean. Asterisks represent adjusted *p* < .05.
**Figure S3.** The latency to enter the light chamber in the light–dark box test. Error bars represent standard errors of the mean.
**Figure S4.** The number of entries into arms in the elevated‐plus maze. Error bars represent standard errors of the mean. Asterisks represent adjusted *p* < .05.
**Figure S5.** The training session in the Barnes maze. (a) Total distance travelled. (b) Latency to escape to the target hole. Error bars represent standard errors of the mean. Asterisks represent adjusted *p* < .05.
**Figure S6.** Activity level and anxiety‐like behaviour of ER animals. (a) Total distance travelled in the open‐field test. (b) Time spent in the centre area in the open‐field test. (c) The number of vertical activities in the open‐field test. Error bars represent standard errors of the mean. Asterisks represent adjusted *p* < .05.
**Figure S7.** The sociality of ER animals (Crawley's social interaction test). (a) Total distance travelled in the trial of mouse cage vs. empty cage. (b) Percent of time staying around the mouse cage in the trial of mouse cage vs. empty cage. (c) Total distance travelled in the trial of novel mouse cage vs. familiar mouse cage. (d) Percent of time staying around the novel mouse cage in the trial of novel mouse cage vs. familiar mouse cage. Error bars represent standard errors of the mean.
**Figure S8.** Stress‐coping strategy of ER animals. Percent of immobile time in the tail suspension test. Error bars represent standard errors of the mean. Asterisks represent adjusted *p* < .05.
**Figure S9.** Single‐nucleotide polymorphism C1473G of Tph2 gene of BALB/cCrSlc mice and a C57BL/6 mouse. The white arrowhead indicates Tph2 product band (523 bp) and black arrowhead indicates genotype‐specific product band (307 bp). NC represents no DNA input.
**Table S1.** Schedule of behavioural test batteries in Experiments 1 and 2.

## Data Availability

All data of this study are available upon request.
