# Supplementary material for: Long‐lasting housing environment manipulation and acute loss of environmental enrichment impact BALB/c mice behaviour in multiple functional domains
Source: Eur J Neurosci. 2022 Feb 23;55(5):1118–40. doi: 10.1111/ejn.15602 (PMC9306724; doi:10.1111/ejn.15602)
Supplement: Supplementary file 1 — Figure S1. EE with an open‐top arena and semi‐EE. (a) Light phase of EE. (b) Dark phase of EE. (c ) Semi‐EE where EE animals were kept during the behavioural test battery. Figure S2. The number of vertical activities in the open‐field test. Error bars represent standard errors of the mean. Asterisks represent adjusted p < .05. Figure S3. The latency to enter the light chamber in the light–dark box test. Error bars represent standard errors of the mean. Figure S4. The number of entries into arms in the elevated‐plus maze. Error bars represent standard errors of the mean. Asterisks represent adjusted p < .05. Figure S5. The training session in the Barnes maze. (a) Total distance travelled. (b) Latency to escape to the target hole. Error bars represent standard errors of the mean. Asterisks represent adjusted p < .05. Figure S6. Activity level and anxiety‐like behaviour of ER animals. (a) Total distance travelled in the open‐field test. (b) Time spent in the centre area in the open‐field test. (c) The number of vertical activities in the open‐field test. Error bars represent standard errors of the mean. Asterisks represent adjusted p < .05. Figure S7. The sociality of ER animals (Crawley's social interaction test). (a) Total distance travelled in the trial of mouse cage vs. empty cage. (b) Percent of time staying around the mouse cage in the trial of mouse cage vs. empty cage. (c) Total distance travelled in the trial of novel mouse cage vs. familiar mouse cage. (d) Percent of time staying around the novel mouse cage in the trial of novel mouse cage vs. familiar mouse cage. Error bars represent standard errors of the mean. Figure S8. Stress‐coping strategy of ER animals. Percent of immobile time in the tail suspension test. Error bars represent standard errors of the mean. Asterisks represent adjusted p < .05. Figure S9. Single‐nucleotide polymorphism C1473G of Tph2 gene of BALB/cCrSlc mice and a C57BL/6 mouse. The white arrowhead indicates Tph2 product band (523 bp) a [file EJN-55-1118-s001.pdf]

Supplementary Methods

Behavioral experiments

General Health and Neurological Examination

In the righting reflex test, animals were lifted up and landed on the floor from their dorsal side. When righting reflex was normal, animals turned their body and landed from their ventral side. In the whisker twitch and ear twitch test, animals' whiskers or ears were touched gently by a cotton applicator. When twitch was normal, animals' whiskers or ears moved quickly. In the reaching test, animals were lifted up by gripping their tails. When reaching was normal, animals reached for nearby objects which they could see. The wire-hang test was performed with a wire mesh box (O'HARA, Tokyo, Japan). The wire mesh (W 100mm, D100mm, 300mm above the floor) was inverted after animals gripped it, and the latency to fall was recorded (max 60 seconds). Grip strength was measured by a Newton scale with a small wire mesh (W 35 mm, D 43 mm, O'HARA, Tokyo, Japan). Animals gripped the mesh by their forelimbs, and they were gently pulled with their tails until they could not grip it. The best score from three trials was recorded. Epilepsy test was performed with the experimenter juggling a bunch of keys for 2 seconds above animals to test the possibility of seizures (acute convulsion, rigidity or faint) caused by loud sounds.

Crawley's Social Interaction Test

The walls of chambers were transparent and had holes (W 50 mm, D 30 mm) which allowed animals to move freely among the three chambers. Two small wire cages (the shape of bottom was 1/4 circle of 100 mm radius, H 100mm) for stranger male C57BL/6N mice (seven-

eight weeks of age) were placed in the inner left corner of the left chamber and in the inner corner of the right chamber, respectively. Chambers were illuminated at 6 lux. Subject animals were positioned in the center chamber when the experiments were started. Subject animals were allowed to move freely during the test. The total distance traveled and time spent in the proximity of cages (60 mm around cages) by the tested animal was recorded.

Prepulse-Inhibition Test

Animals were constrained in a transparent cylinder (25 mm in diameter) during the test. The box was illuminated at 3000 lux with under 50 dB background white noise. First, animals were habituated to the test cylinder and box for 300 seconds. Next, acoustic startle responses were measured by acoustic stimuli in the startle stimulus trials. Each stimulus was presented four times (total 16 stimuli) and average startle responses were recorded. Intervals between each stimulus were randomly selected from 5, 10, 15, 20, 25 seconds to prevent animals from predicting the timing of stimuli. Subsequently, prepulse inhibition of acoustic startle responses was measured in the prepulse inhibition trial. Each pair was presented four times (total 16 pairs) and average startle responses were recorded. The prepulse stimuli sound lasted 100 msec before the startle stimulus. Intervals between each paired stimulus were randomly selected from 5, 10, 15, 20, 25 seconds. Amplitudes were sampled from 400 msec following startle stimuli and averages of amplitudes of same four stimuli were recorded. The percent of prepulse inhibition was calculated as follows; PPI (%) = (amplitude to 120 dB in the startle stimulus trial - amplitude in the prepulse inhibition trial) / amplitude to 120 dB in the startle stimulus trial × 100.

Barnes Maze

The arena was 760 mm above the floor and illuminated at 1000 lux. A black escape box (W 160 mm, D 120 mm, H 60 mm) filled with white paper bedding material was placed under one of the holes as the target hole. The position of the target hole was fixed during individual tests and positions of target holes were randomly chosen for different individuals. Four spatial cues (a big blue rectangular, yellow sphere, red quadrangular pyramid, and black coil) were hung from the ceiling in the four corners of the experimental room. The arena was rotated every day to avoid animals using olfactory or proximal cues in the arena. The training session consisted of 16 trials (1 trial/day, 5 minutes). Animals were placed in the center area inside of white opaque cylinder (110mm in diameter, H 168 mm), and when the experiments started, the cylinder was taken out. The starting point was randomized within the center area. Animals moved freely on the arena during the test. The total distance traveled and the latency to enter the targeted hole was recorded. When animals did not enter the target hole within 5 minutes, they were guided to the target hole and left there for 30 seconds. The latency was recorded as 300 seconds in this case. One day after the training session, the first probe test for 5 minutes was conducted in the absence of the target hole, to confirm that the animals were only guided by the distal spatial cues. After the first probe test, one training trial was conducted as a retraining. A second probe test was conducted one week after the first probe test with the same procedure. Time spent around holes was recorded. Missing values in data were complemented by means of the value of the previous trial and next trial. When missing values were in the data of trial 16, they were complemented by the values of trial 15. If missing values were in sequential trials, the data was excluded from analysis.

Here data had some missing values due to administrative failures in the training session (14 missing points/ total 445 data collecting points), and processed as mentioned above.

Fear-Conditioning Test

On the first day, conditioning was conducted in a test chamber illuminated at 100 lux (W 327 mm, D 250 mm, H 284 mm; O'HARA, Tokyo, Japan). Animals could move freely in the chamber for 8 minutes. First, animals were habituated to the chamber for 120 seconds. Second, Three CS [conditioned stimulus, 30 seconds of white noise (55 dB, 500 - 20000 Hz)) – US (unconditioned stimulus, last 2 seconds of the tone, 0.30 mA electrical foot shock) pairs were presented (120-150 second, 240-270 second, 360-390 second). On the second day contextual test was conducted in the same chamber. Animals could move freely in the chamber during the test for 5 minutes without white noise or foot shock. On the third day, cue test was conducted in a new triangular chamber (333 mm × 333 mm × 333 mm, H 400 mm, O'HARA, Tokyo, Japan). The chamber was illuminated at 10 lux. Animals could move freely in the chamber during the test for 6 minutes. First, animals were habituated to the chamber 180 seconds (pre-cue phase). Second, 180 seconds of 55 dB white noise was presented (180-360 second) without foot shock (cue phase). The percent of immobile time (freezing) was recorded.

SNP Genotyping

Genotyping primers (Zhang et al, 2004):

Tph2 forward primer, (5'TTTGACCCAAAGACGACCTGCTTGCA);

Tph2 reverse primer, (5'TGCATGCTTACTAGCCAACCATGACACA);

C-allele specific primer, (5'CAGAATTTCAATGCTCTGCGTGTGGG);

G-allele specific primer, (5'CAGAATTTCAATGCTCTGCGTGTGGC).

Supplementary Figures and Tables

Figure S1

EE with an Open-top Arena and Semi-EE. (A) Light-phase of EE. (B) Dark-phase of EE. (C) Semi-EE where EE animals were kept during the behavioral test battery.

Figure S2

The Number of Vertical Activities in the Open-Field Test. Error bars represent standard errors of the mean. Asterisks represent adjusted $p < .05$.

Figure S3

The Latency to Enter the light chamber in the Light-Dark Box Test. Error bars represent standard errors of the mean.

Figure S4

The Number of Entries into Arms in the Elevated-Plus Maze. Error bars represent standard errors of the mean. Asterisks represent adjusted $p < .05$.

Figure S5

The Training Session in the Barnes Maze. (A) Total distance traveled. (B) Latency to escape to the target hole. Error bars represent standard errors of the mean. Asterisks represent adjusted $p < .05$.

Figure S6

Activity Level and Anxiety-like Behavior of ER animals. (A) Total distance traveled in the open-field test. (B) Time spent in the center area in the open-field test. (C) The number of vertical activities in the open-field test. Error bars represent standard errors of the mean. Asterisks represent adjusted $p < .05$.

Figure S7

The Sociality of ER animals (Crawley's social interaction test). (A) Total distance traveled in the trial of mouse cage vs. empty cage. (B) Percent of time staying around the mouse cage in the trial of mouse cage vs. empty cage. (C) Total distance traveled in the trial of novel mouse cage vs. familiar mouse cage. (D) Percent of time staying around the novel mouse cage in the trial of novel mouse cage vs. familiar mouse cage. Error bars represent standard errors of the mean.

Figure S8

Stress Coping Strategy of ER animals. Percent of immobile time in the tail-suspension test. Error bars represent standard errors of the mean. Asterisks represent adjusted $p < .05$.

Figure S9

Single-Nucleotide Polymorphism C1473G of Tph2 Gene of BALB/cCrSlc Mice and a C57BL/6 Mouse. The white arrowhead indicates Tph2 product band (523 bp) and black arrowhead indicates genotype-specific product band (307 bp). NC represents no DNA input.

Table S1

Schedule of behavioral test batteries in Experiment 1 and 2.

Figure S1

EE with an Open-top Arena and Semi-EE

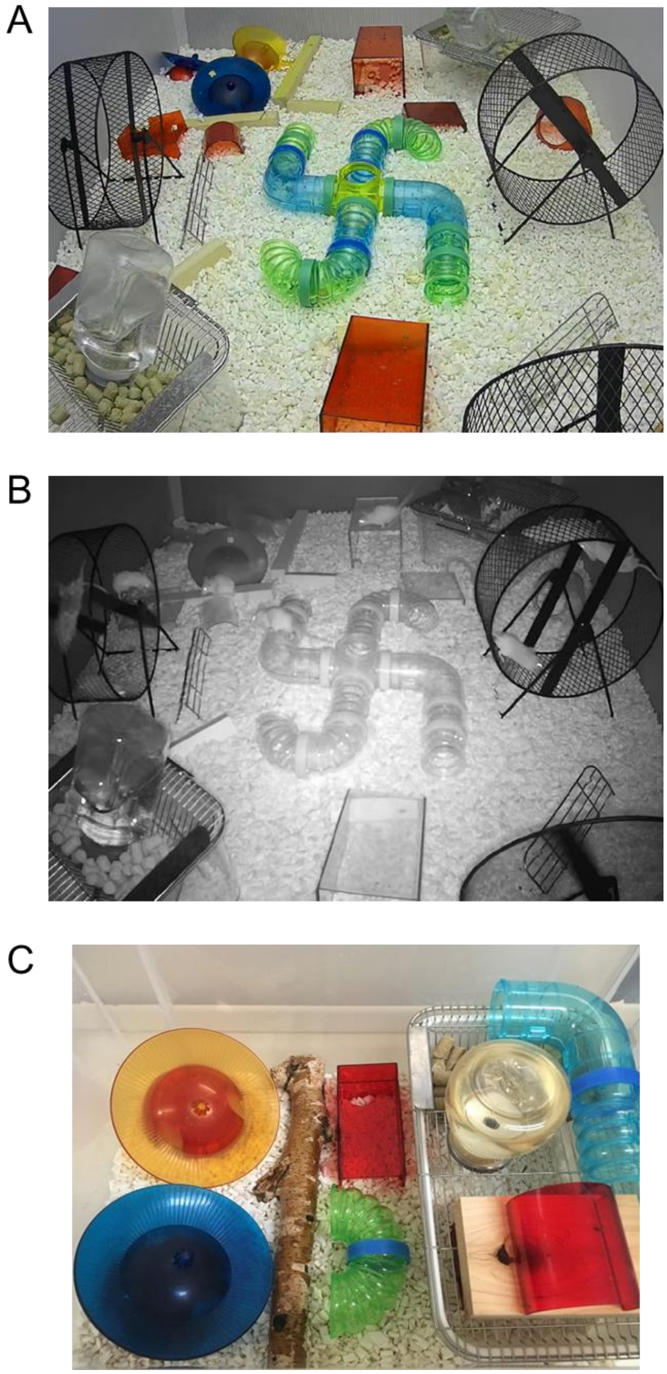

Figure S2

The Number of Vertical Activities in the Open-Field Test

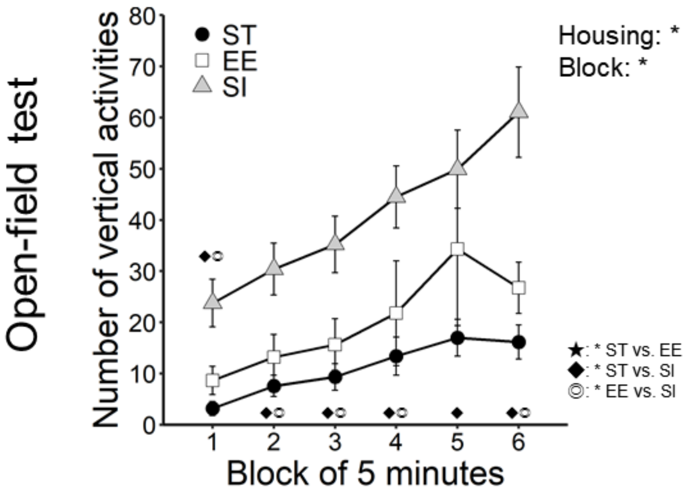

Figure S3

The Latency to Enter the light chamber in the Light-Dark Box Test

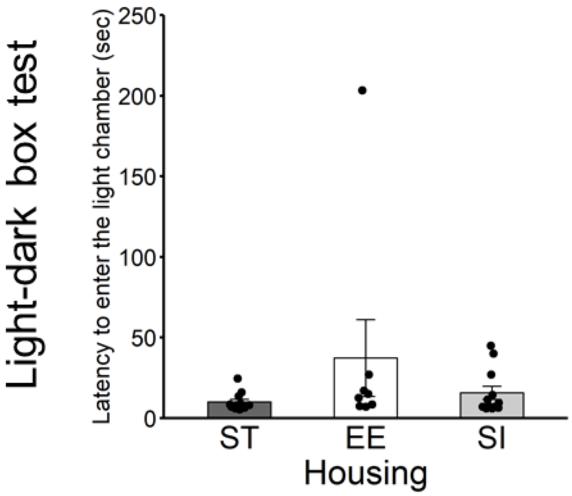

Figure S4

The Number of Entries into Arms in the Elevated-Plus Maze

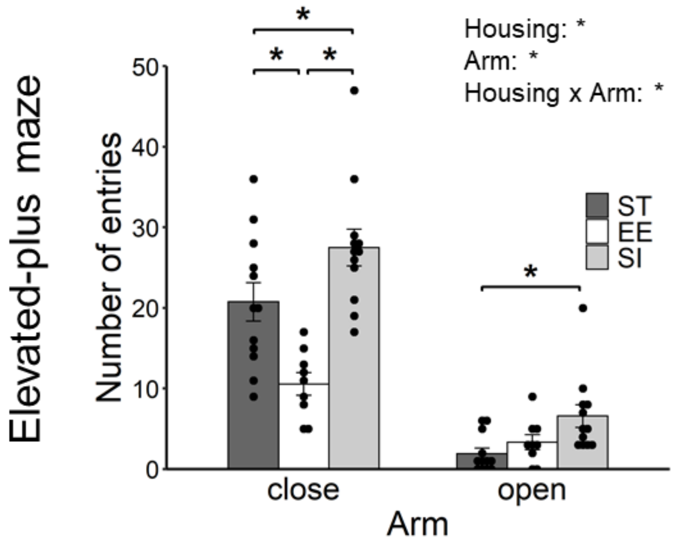

Figure S5

The Training Session in the Barnes Maze

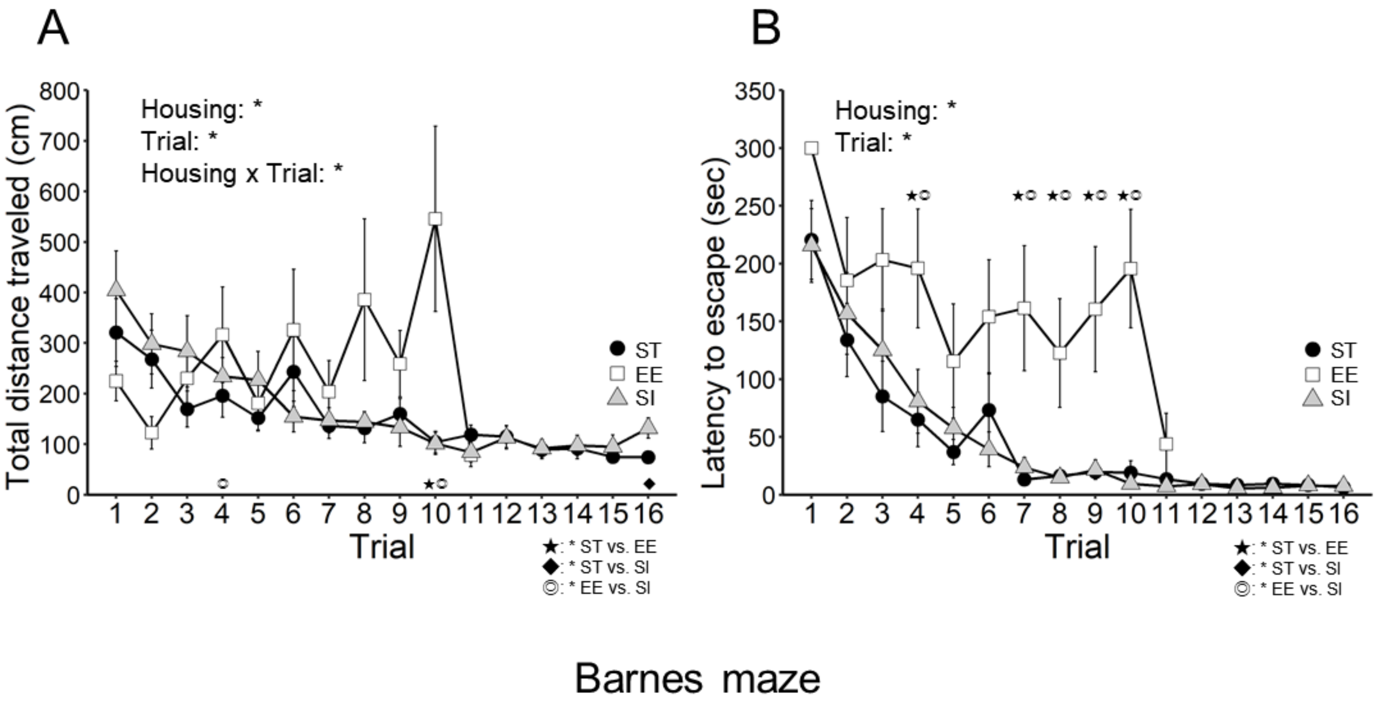

Note: The total distance traveled in the training session is shown in panel A. In trial 1 - 11 (12 ST animals vs. 7 EE animals vs. 11 SI animals), a 3 (housing; ST, EE, and SI; between-animal) × 11 (trial; within-animal) ANOVA was conducted. The main effect of housing was significant [F(2, 27) = 4.01, p = .030, η_p^2 = .229]. The subsequent analysis revealed that there were significant differences between ST and EE group (p = .013, adjusted p = .040, r = .45) and between EE and SI group (p = .022, adjusted p = .044, r = .42). EE animals showed longer total distance traveled than ST and SI animals. The main effect of trial was significant [F(10, 270) = 3.03, p = .001, η_p^2 = .101]. The interaction between housing and trial was significant [F(20, 270) = 3.17, p < .001,

η_p^2 = .190]. The subsequent analysis revealed that EE animals showed longer total distance traveled than ST or SI animals in trial 4 and 10. In trial 12 - 16 (12 ST animals vs. 11 SI animals), a 2 (housing; ST and SI; between-animal) × 5 (trial; within-animal) ANOVA was conducted. The main effect of housing was not significant [F(1, 21) = 1.03, p =.321, η_p^2 = .047]. The main effect of trial was not significant [F(4, 84) = 0.91, p = .464, η_p^2 = .041]. The interaction between housing and trial was not significant [F(4, 84) = 1.01, p = .407, η_p^2 = .046].

The latency to escape to the target hole in the training session is shown in panel B. In trial 1 - 11 (12 ST animals vs. 7 EE animals vs. 11 SI animals), a 3 (housing; ST, EE, and SI; between-animal) × 11 (trial; within-animal) ANOVA was conducted. The main effect of housing was significant [F(2, 27) = 14.63, p < .001, η_p^2 = .520]. The subsequent analysis revealed that there were significant differences between ST and EE group (p < .001, adjusted p < .001, r = .69) and between EE and SI group (p < .001, adjusted p < .001, r = .67). EE animals showed longest latency than ST and SI animals. The main effect of trial was significant [F(10, 270) = 17.98, p < .001, η_p^2 = .400]. The interaction between housing and trial was not significant [F(20, 270) = 1.06, p = .390, η_p^2 = .073]. In trial 12 - 16 (12 ST animals vs. 11 SI animals), a 2 (housing; ST and SI; between-animal) × 5 (trial; within-animal) ANOVA was conducted. The main effect of housing was not significant [F(1, 21) = 0.96, p =.339, η_p^2 = .044]. The main effect of trial was not significant [F(4, 84) = 0.84, p = .506, η_p^2 = .038]. The interaction between housing and trial was not significant [F(4, 84) = .0.83, p = .512, η_p^2 = .038].

Figure S6

Activity Level and Anxiety-like Behavior of ER animals

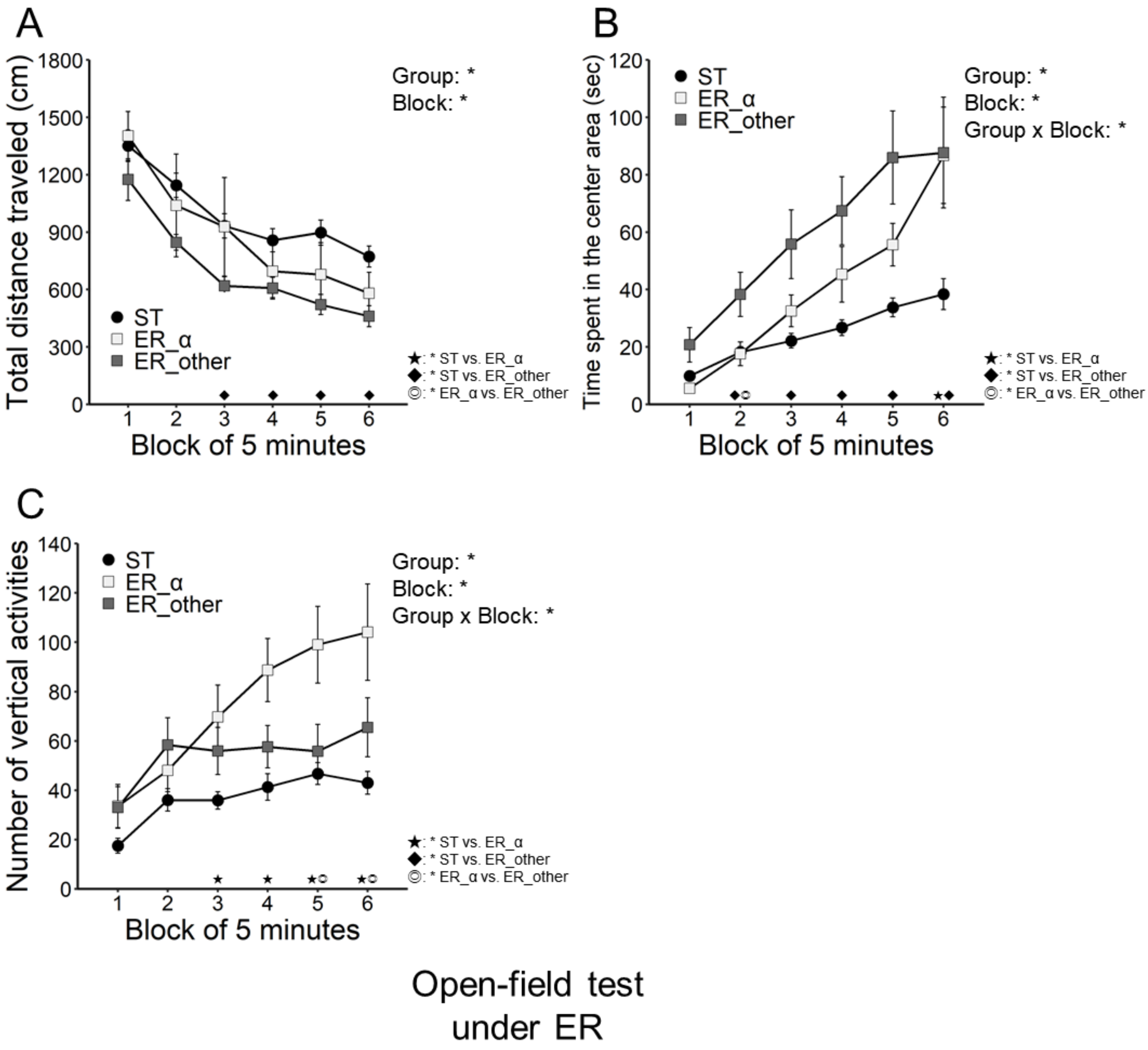

Figure S7

The Sociality of ER animals

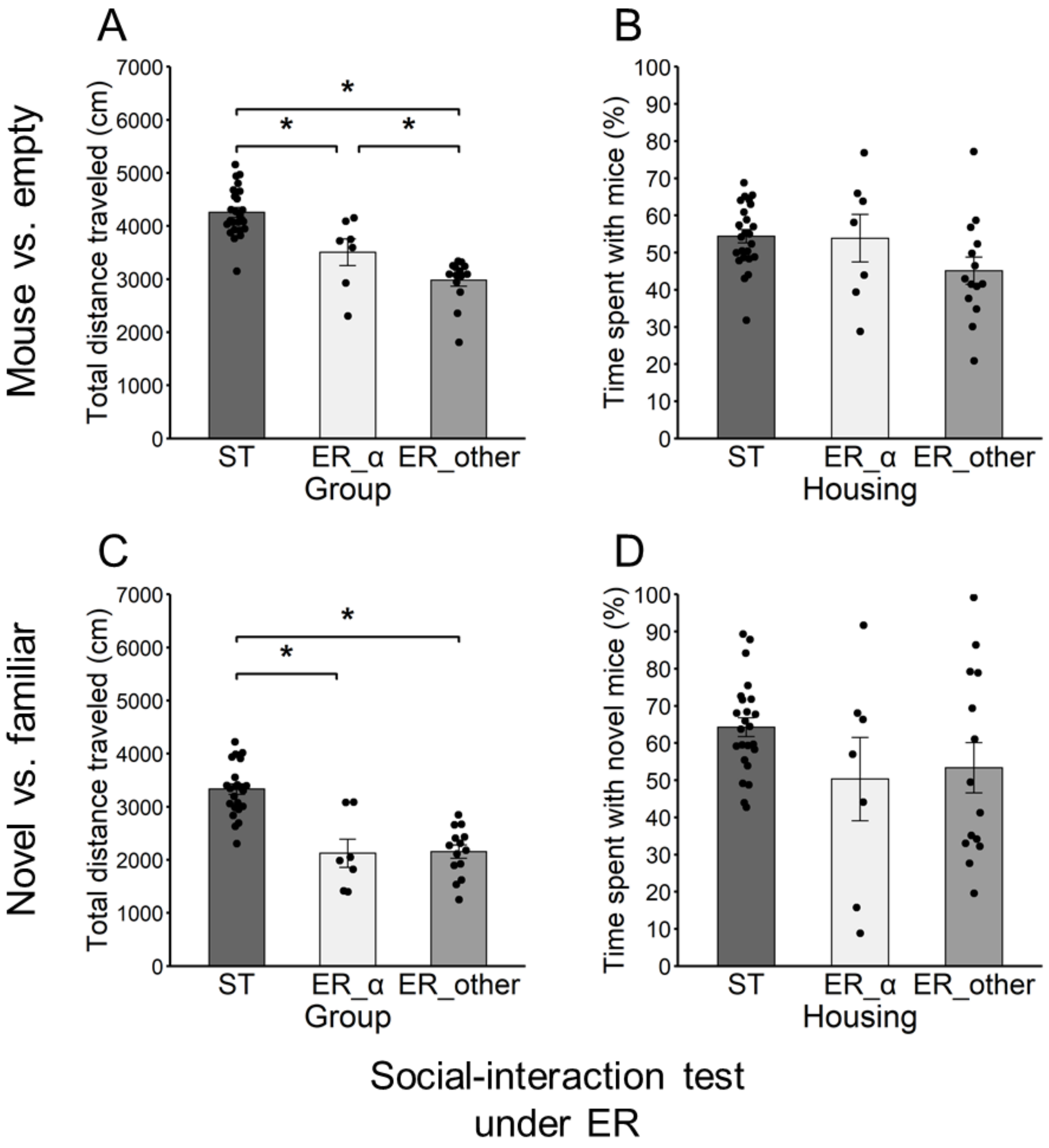

Figure S8

Stress Coping Strategy of ER animals

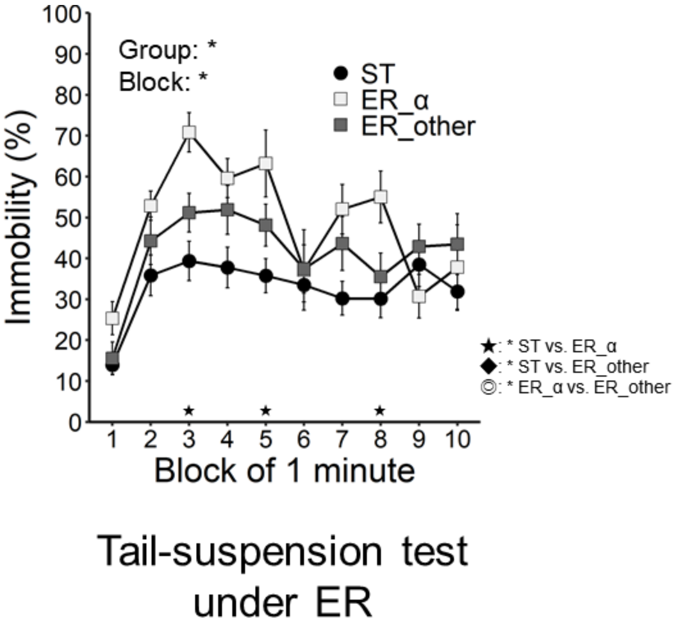

Figure S9

Single-Nucleotide Polymorphism C1473G of Tph2 Gene of BALB/cCrSlc Mice and a C57BL/6 Mouse

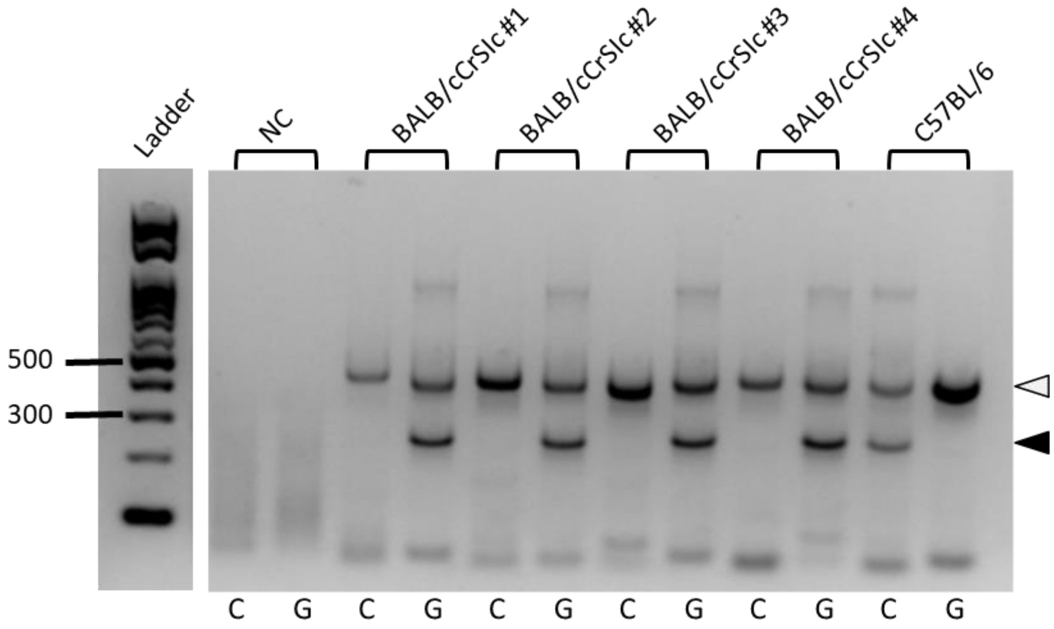

Table S1

Schedule of behavioral test batteries in Experiment 1 and 2

	Experiment 1	Experiment 2
Health check and/or handling	0-5	0
Open field	2-6	1
Y maze	3-8	
Elevated-plus maze	6-9	
Rotarod	7-29	
Hot plate	10-29	
Social interaction	11-20	4-8
Porsolt swim	14-35	
Prepulse inhibition	19-31	
Light-dark box	20-93	
Barnes maze	32-76	
Fear conditioning	53-79	
Tail suspension	50-90	11

(day)
